# Genetics and genomic medicine in Tunisia

**DOI:** 10.1002/mgg3.392

**Published:** 2018-04-16

**Authors:** Houda Elloumi‐Zghal, Habiba Chaabouni Bouhamed

**Affiliations:** ^1^ GeneDx Gaithersburg MD USA; ^2^ University Tunis El Manar Tunis Tunisia

## Abstract

Genetics and genomic medicine in Tunisia.

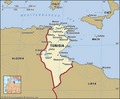


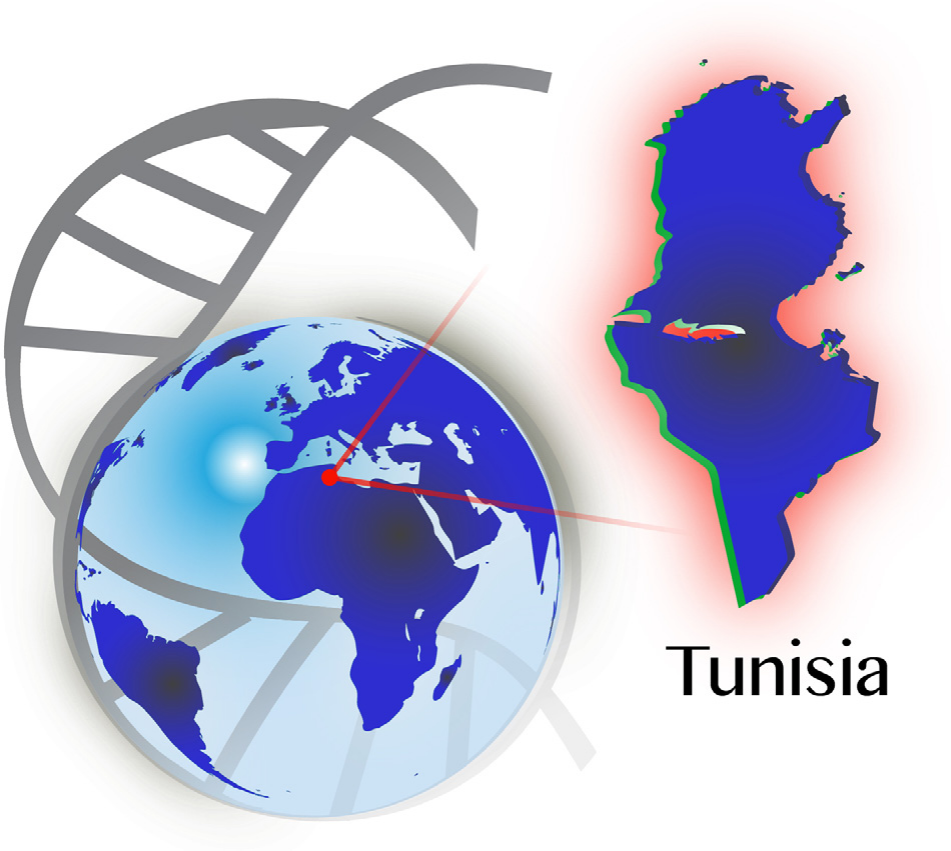

A Carthagian beauty who is still, from the Andalusia time, carries in her voice, a flowing hymn. O Tunisia, the dreams, you are the guard of arts, melodies, and scents. Farooq Juwaida

## 1 GENERAL OVERVIEW, DEMOGRAPHIC STATISTICS, ECONOMY, AND EDUCATION

**Figure 1:**
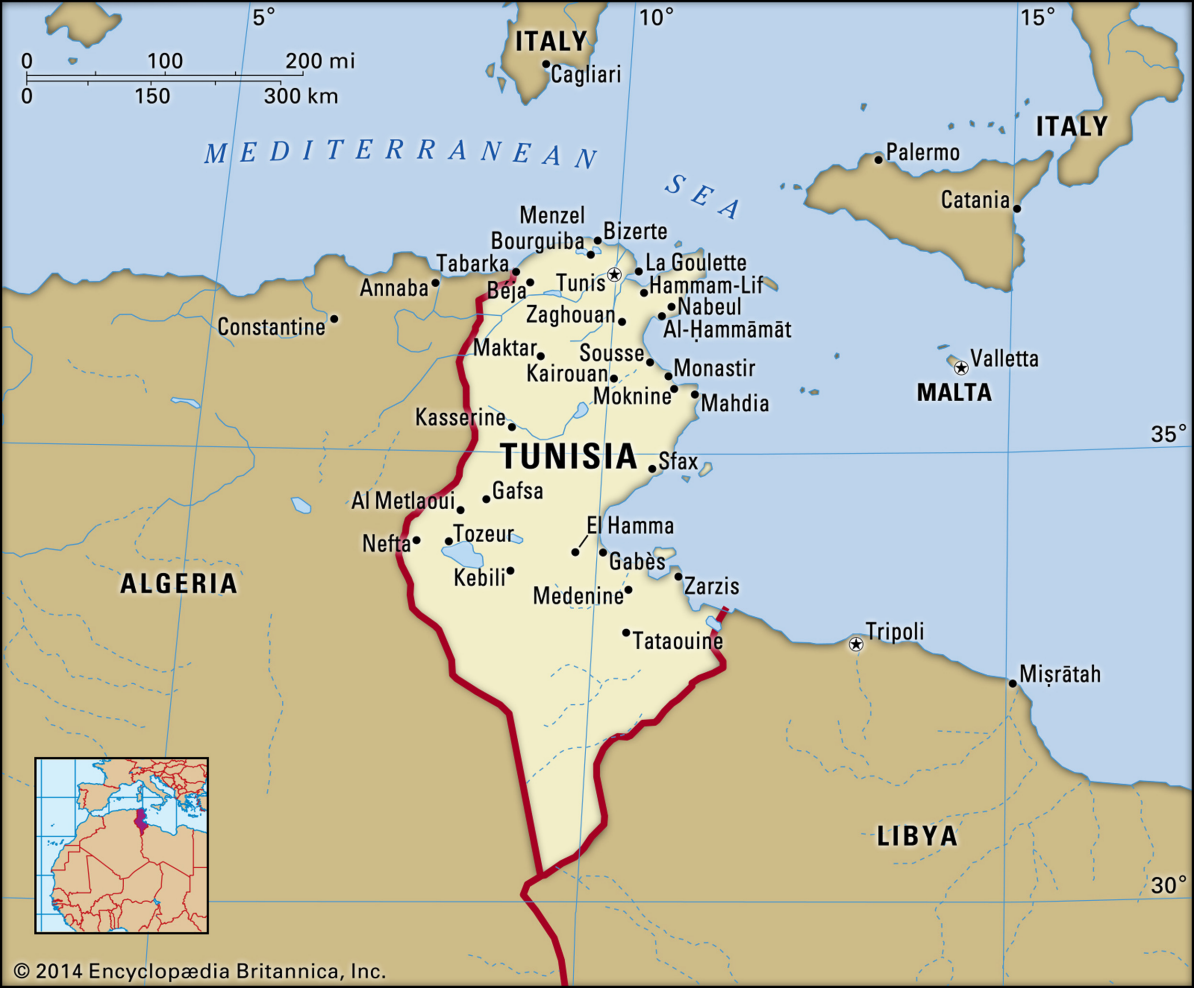
Map of Tunisia, boundaries and cities (https://www.britannica.com/media/full/609229/61574)

Tunisia is situated on the northern coast of Africa, bordered by Algeria to the west, Libya to the southeast and the Mediterranean Sea along its northern and eastern sides (Figure 1). It is the smallest country in North Africa and covers 163,610.0 sq. km; slightly larger than Georgia (World bank #92), with 1,148 km of coastline. Tunisia is divided into 24 governorates; the capital Tunis is the smallest and the most populated.

As of 1 July 2015, the number of inhabitants was 11,154,370 (5,557,966 males and 5,596,404 females), 2.94 times higher since independence (3.78 millions in 1956). About 45% of the population is represented by individuals between the ages of 25 and 54, with a median age of 32.4 years. The growth rate is estimated at 0.86%. The majority of the population occupies the northern and coastal region of the country, with 66.8% living in urban areas.

Because of its strategic location at the crossroads of Europe, Africa and the Middle East, Tunisia witnessed the succession of many civilizations throughout its history. Its population is ethnically heterogeneous, with the presence of the indigenous Berbers together with people from the invading civilizations (Phoenicians, romans, Arabs, and others) as well as from migration.

Formal Arabic is the official language, however, natives speak a dialect of Tunisian Arabic and Shilha is still used by Berbers. French is the language used in most of the institutions; it plays an important role in education and the press. English is currently considered the second foreign language and it is now introduced at the elementary school level. The overwhelming majority of Tunisians (98%) are Muslim, the rest are Christians, Jews, and others.

The Tunisian family size average is 4.1; it is getting smaller in big cities (General population census 2014, National Institute of Statistics). There is prevalence of consanguineous marriages and geographic endogamy, mainly in the more rural areas. Consanguinity rate ranges from 20.1% to 39.33% (Ben Arab, Masmoudi, Beltaief, Hachicha, & Ayadi, 2004; Riou, el Younsi, & Chaabouni, 1989).

Based on the 2014 statistics, Tunisia is classified in the high development category with a human development index (HDI) value of 0.721 compared to 0.486 in 1980, an increase of 48.4%. Furthermore, the gross national income per capita increased by 101.8%. However, this development is unevenly distributed and is much higher in the eastern coastal regions. Regional inequities are considered the main barrier to socioeconomic development, with more developed coastal regions and an impoverished interior. As per 2010 data, 15.5% of the population is living below the poverty line. Such gaps between the different regions became more evident after the 2011 revolution (Hermassi, 2013).

A few years after the revolution, Tunisia's economy remains weak, with a high unemployment rate (17.6%); particularly among young people and university graduates where it reaches 37.6%. This situation led to labor emigration mainly to European countries. Bilateral labors agreements were signed with a number of these countries with the expectation that these young workers will later return home. Unfortunately, the lack of job creation in Tunisia together with skill mismatches and the imposed restriction of immigration by European countries created a massive illegal migration flow (mainly to Europe). Furthermore, this situation worsened following the Civil war in Libya as around 1.8 million Libyans have fled to Tunisia.

Just after the independence, our first president, Habib Bourguiba, enacted a law that allows all inhabitants to have access to free schools regardless of their religion, sex or race. Later, in 1991, the first 9 years of schooling became compulsory and education was established as a national priority. However, the government does not mandate preschool, and most of the facilities are private.

In 2008, the World Bank reported that 96.79% of people between the ages of 15 and 24 were literate, which provides a strong foundation of hope for the future of literacy in Tunisia. The rate of illiteracy among women was somewhat higher than that among men.

The 2014 data showed that 32.8% of adult Tunisian women have reached at least a secondary level of education compared to 46.1% of their male counterparts. The report showed that 31.3% of parliamentary seats are hold by women, compared to only 14% in Arab states. However, female participation in the labor force is much lower compared to men (25.1 versus 70.9%).

The education system in Tunisia is recognized as one of the most modernized in the region. Several laws were instated to help with the integration of special needs students. However, not enough specialized centers to help patients with developmental and cognitive delays are offered and the increase in the number of integrative schools was paralleled by an increase in enrolled students. Furthermore, there is a lack for trained professionals that can meet the needs of these patients. UNICEF data showed that 90% of teachers in integrative schools are not prepared to teach special education and did not receive any formal and specific training (Shaub, 2016).

## 2 TUNISIA: FROM THE STONE AGE TO THE PRESENT ERA

Evidence of Paleolithic settlement was found in different archeological sites in Tunisia, one of the oldest being Sidi Zin in the Northwest of the country (Gragueb, 1980). Excavated by Gobert in 1950, the site of Sidi Zin is located 11 km south of the city of Kef, and is particularly known for its Acheulian levels. Artifacts from the Mousterian level, characteristic of the middle Paleolithic, were also found and included small bifaces, sidescrapers, and thick points (Aouadi‐Abdeljaouad & Belhouchet, 2012). During recent excavations by Tunisian and British researchers near Tozeur, in the southwest of the country, flint tools—among other artifacts—were discovered and reported to be the oldest evidence of human activity in Tunisia (92,000‐years old). Bones indicating the presence of savannah animals and therefore fresh water were unearthed.

The excavation of the Aïn El‐Guettar Mousterian site, located approximately 15 km southeast of Gafsa, yielded a faunal assemblage dominated by bovids and equids. The site was associated with a lithic industry with Levallois technology (Aouadi‐Abdeljaouad & Belhouchet, 2008; Gruet, 1954). The same site also showed the presence of the Aterian, another Middle Stone age culture. The Aterian industry is based mostly of flakes, Levallois techniques, scrapers and racloir tools. The earliest Aterian sites are believed to be well over 40,000‐year old (Frigi, Cherni, Fadhlaoui‐Zid, & Benammar‐Elgaaied, 2010). Furthermore, stone tools were found in the northern and central coastal areas of Tunisia going back to the Aterian civilization.

Small flint tools of around 20,000 BC were attributed to a new culture that became the precursor to North Africa's Iberomaurusian civilization (18,000 to 8,000 BC). Small retouched blades, backed bladelets, simple endscrapers, geometrical pieces, among other artifacts were found in the site of Ouchtata, northwest of Tunisia.

The Iberomaurusian people belonged to the Mechtoid anthropological type, also known as Mechta el‐Arbi or Mechta‐Afalou that inhabited the region during the late Paleolithic and Mesolithic. The Iberomaurusian culture was thought to have emerged either as a result of the migration of Cromagnoids from the Iberian Peninsula, or from the local Aterian culture (Ferembach, 1985).

**Figure 2:**
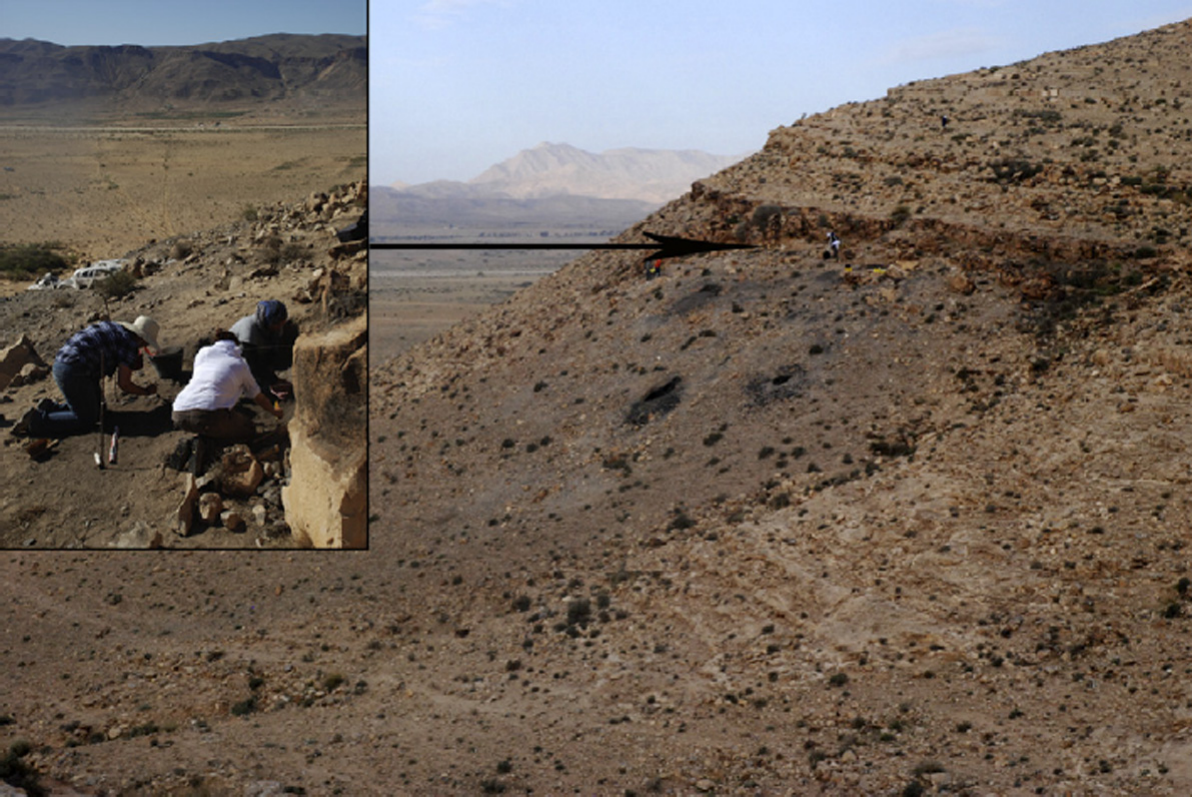
Overview of the open‐air Capsian site “EL‐Mekta”

During the Holocene, the Capsian civilization covered the period between 10,000 and 7,500 BP. This culture was discovered during the excavation of the site of El‐Mekta, located by Boudy in 1906 on the eponymous hill, 10 km north of Gafsa in southwest Tunisia (Morales et al., 2015) (Figure 2). Archeologists were able to find stone and bone tools, shell beads and decorated bones, ostrich shell containers, as well as stylized women's heads carved from limestone. The Capsians left shell heaps near their dwellings, indicating that snails were an important part of their diet. Evidence for hunting, fishing, food processing and cooking was also abundant. Remains of Aleppo pine as well as Acorns were the commonly detected in the plant assemblage recovered from site El‐Mekta (Morales et al., 2015).

Capsians might be the immediate ancestors of Berbers (Rahmani, 2004). The later Neolithic people possibly introduced the Afro‐Asiatic languages (Arredi et al., 2004; Sanchez‐Mazas, 2000). The indigenous Berbers of Tunisia are called “Amazigh” which means “free people”.

During the historical period, the first important invasion was that of the Phoenicians, ancestors of present‐day Lebanese, who used their maritime expertise to establish trading posts in many cities across the Mediterranean (Zalloua et al., 2008). In the eigth century B.C (around 814 BCE), after she fled from her brother Pygmalion, king of Tyre (what is today Lebanon), Elissa, also known as “Dido”, and her followers established the colony of “Kart‐Hadasht” (latin Carthago) known as “Carthage” and meaning “New City”. Carthage later became the greatest naval power of the Mediterranean. The temple of Eshmun, the Punic god of healing, was at that time built on the peak of Byrsa Hill, the highest point in the city of Carthage. During the Roman Empire, a cathedral (known now as Saint Louis Cathedral) was built atop of its ruins.

All we know about the Phoenicians is through Greek and Roman sources, since all evidence was destroyed and the Carthaginian literature was lost. Recently, a Punic burial crypt was accidentally discovered by gardeners while planting a tree at the front of the National Museum of Carthage, situated on Byrsa Hill, at the site of the Phoenician acropolis (http://www.inp.rnrt.tn/index.php?option=com_content&view=article&id=75:jeune-homme-de-byrsa&catid=3: activites&Itemid=7&lang=en). The skeletal remains of a young man along with funerary goods were found, all dated to the late sixth century BC (Morel, 2011). DNA extracted from a small bone sample was used for the first complete ancient Phoenician mitochondrial genome study. An international team of researchers showed that the mitochondrial genome of this “young man of Byrsa” belongs to the haplogroup U5b2c1, a European derived haplotype absent in North Africa and in Lebanese (Matisoo‐Smith et al., 2016). Data from other remain throughout the Mediterranean will help us understand the origin of Phoenicians and trace their migrations.

Following the third Punic war (149–146 BCE) between the Romans and Phoenicians, the Roman Empire became the most powerful force in the Mediterranean region and the region was completely Latinized and Christianized. The Vandals, a Germanic tribe, later succeeded Romans; they reigned for almost a century before falling to the armies of Byzantines in 533 AD (Brett & Fentress, 1997).

By the end of the seventh century A.D, the Islamic invasion of North Africa was carried by armies from the Arabian Peninsula (Lapidus, 2002). They founded the city of Kairouan, about 90 miles south of Carthage, and they spread Islam as well as the Arabic language in the region. Fatimids’ invasion then followed in the 11th century AD. The Ottomans then ruled Tunisia for over 400 years.

During the 16th and 17th centuries, epidemic waves, cricket invasions, and starvations led to the death of thousands of individuals. Tunis was then affected by the plague for 19 years; nearly 1,000 individuals died because of epidemics of plague, cholera and typhus in 1918.

As the Ottoman Empire declined, the Berlin Congress of 1878 convened to discuss the future of the North African provinces. As a result, Britain acquiesced to France's control of Tunisia and in exchange took over Cyprus. In 1881, the French sent their troops to invade Tunisia and imposed the treaty of Bardo. In 1883, the Bey of Tunis signed an agreement that made Tunisia a French protectorate. It is not until 1956 that Tunisia got her independence from France and became a republic under the leader Habib Bourguiba. On 25 July, 1957, a republic was declared and Bourguiba became the first president of Tunisia. In November 1987, his appointed first prime minister “Ben Ali” removed him from power after a bloodless coup. The latter stayed in power for 23 years until the Tunisian revolution and the collapse of the regime on 14 January 2011. On 27 January 2014, the new president signed the latest approved constitution replacing the one instated in 1959 and amended in 1988 and 2002. The country is still recovering after this political unrest and the violence threatening economic progress.

## 3 POPULATION DIVERSITY

Although Berbers are the autochthonous inhabitants of the country, the major historical settlements had a crucial impact on the genetic structure and diversity of the current population. As a result, the latter derives its origins from Phoenicians, Romans, Vandals, Byzantines, Africans, Arabs, Turks, Europeans as well as immigrants. Despite all these historical admixtures, some genetic isolates still exist. The best example is the inhabitants of Jerba, an island in the South‐East of Tunisia (Loueslati et al., 2006).

To determine the degree of heterogeneity of this population, analysis of 16 Alu and 3 Alu/STR compound systems was carried on 268 healthy unrelated individuals originating from the north‐center and the south regions. Similar levels of gene diversity were found in both groups. Interestingly, the northern sample showed higher frequencies of Berber and sub‐Saharan African‐specific combinations than the southern sample (El Moncer et al., 2010). An indication of sub‐Saharan gene flow was also shown by a previous mtDNA haplotype data (Turchi et al., 2009).

Trans‐Saharan trade is believed to be the main source of the African flow to the region, although few came from the Arab invasions and from Europe during the colonization of the Maghreb (Northwest Africa). South Tunisia constituted the gateway for Arab tribes invading North African cities. Archeological and historic records suggest that such migration is ancient and could be traced back to 9000 years BP, characterized by an ethnic contribution from present‐day Sudan (El Moncer et al., 2010).

According to the official census in 2014, the population living in the south of the country comprises Berbers, Black people, Jews, and Arabs. Most of the studies agreed that the southern Tunisians are native Berbers that were later Arabized during the Arab invasions in the 11th century (Hajjej et al., 2006; Hajjej, Sellami, et al., 2011).

In an effort to trace the origin of the southern population, a recent “anthropological” study of 250 unrelated Southern Tunisians was conducted using HLA typing, commonly used to track human migrations (Fernandez Vina et al., 2012). Data were compared to those of other Tunisians, Middle Eastern Arab‐speaking individuals along with Mediterranean and sub‐Sahara African populations. DRB1*07:01–DQB1*02:02 was found to be the most frequent haplotype in Southern Tunisians (18.02%). This haplotype was also present in Tunisian Berbers (16.03%) (Hajjej, Almawi, Hattab, El‐Gaaied, & Hmida, 2016). In fact, the HLA class II allele DRB1*07:01 was present at 22.06% in the southern population, and was detected at high frequencies in Tunisian Berbers (17.6%) and in Ghannouchians (28.7%) occupying a village situated at the eastern south of Tunisia and characterized by high endogamy, behaving like an isolate (Hajjej, Hajjej, et al., 2011).

Following the Arab invasion of Tunisia, Berbers migrated to the south and populated the mountainous regions to escape persecution. This resulted in a low admixture between them and the Arab tribes. This fact was further confirmed by the HLA typing data that demonstrated the low contribution of Arabs to the southern genetic pool (Hajjej et al., 2016).

A Y‐chromosome lineage analysis was carried on 94 individuals from three Northern and southern Berber‐speaking isolates. Two of the isolates exclusively carried the haplogroup E1b1b1b, also found in other Berber‐speaking groups in North Africa. These two groups lived in the mountains, and historically escaped persecution by the Arab tribes “Banu Hilal and Banu Soulaym” around 1048 AD. Haplogroup J (subtype J1e), previously associated with the Islamic expansion, was found in 31.4% of the third Berber group, living in the plains, indicative of genetic admixture and gene flow from the Near East. However, and in contrast to the mtDNA studies findings, the Y‐chromosome data did not show any evidence of sub‐Saharan African paternal lineages (Fadhlaoui‐Zid et al., 2004, 2011).

Another study investigating the profile of HLA class I and class II genes was carried on a total of randomly selected 376 unrelated Tunisians originating from the North, South and center of the country. A*02:01(16.76%) and DRB1*07:01 (19.02%) alleles, both present at high frequency in Berbers, were found to be the most frequent alleles. Common haplotypes found in Tunisians were also seen in Western Mediterranean populations. There was no evidence of an effect of the Arab invasions (7th and 11th centuries AD) on the genetic makeup of the Tunisians including the autochthonous Berbers. This suggests that no or minimum admixtures occurred between Berbers and Arab tribes. One possible explanation can be the modest number of Arab invaders during the 7th century. Moreover, establishment of settlements did not follow this invasion. Haplotype studies showed that the current inhabitants of the country are closely related to Iberians (Basques and Spaniards) as well as to North Africans but not to Eastern Arabs (Hajjej, Almawi, Hattab, El‐Gaaied, & Hmida, 2015).

Following the Bizerte crisis with France and the 6‐day War of 1967, thousands of Jews fled the country. Nowadays, Tunisian Jews constitute less than 0.1% of the total population (1,500); they are mainly clustered in the island of Jerba. Although they constitute a distinct branch, North African Jews showed a significant relatedness to European and Middle Eastern Jews. The Jews from Jerba and the rest of Tunisia were highly endogamous, with very low level of admixture with Arab and Berber inhabitants as shown by Y‐chromosome studies (Campbell et al., 2012). It is believed that the first evidence for Jews in North Africa goes back to 312 BCE when King Ptolemy of Egypt settled Jews in the cities of Cyrenaica in current‐day Libya. Historians also reported that in 70 CE, the Roman Emperor Titus destroyed the Great Temple of Jerusalem leading to the deportation of 30,000 Jews to Carthage in present‐day Tunisia. Others traced the origins of Jews in Tunisia to Andalus and Levant (Lucette & Abraham, 1991). Following the Arab invasion, some Jewish communities remained in the country but were subject to civil and religious suppression resulting in the high degree of endogamy.

## 4 HEALTHCARE COVERAGE IN TUNISIA

The newly drafted constitution in 2014 placed national health care at a higher priority and “proclaimed health as a human right and required national healthcare coverage for the poorest of Tunisians” under Article 38.

Since its independence in 1956, Tunisia made health care free for all through a government‐funded system. Four years later, a social protection system with a health insurance scheme and a subsidized care was implemented.

In 1996, the implementation of “large scale reform” was intended to improve coverage and accessibility to healthcare (Achour, 2011). Several insurance plans covering different professional groups were merged under the “social security Fund”. The benefit package was also extended to include providers in the private sector. Furthermore, an optional complementary health insurance managed by mutual health insurance companies was also introduced. Following the 2004 reform, Tunisia's National Health Insurance Fund “CNAM” (Caisse nationale d'Assurance maladie) was created, aiming to provide universal health coverage for those affiliated with national insurances (CNSS and CNRPS). Coverage was extended to include inpatient and outpatient services provided by the private sector. In late 2007, CNAM introduced a reimbursement scheme also called the “two sector scheme”, where beneficiaries can use either public or private providers but are required to first pay then request reimbursement from the CNAM (Makhloufi, Ventelou, & Abu‐Zaineh, 2015). Such reform has raised the coverage from 54.6% in 1995 to 88% in 2008 (Abu‐Zaineh, Romdhane, Ventelou, Moatti, & Chokri, 2013).

The current health insurance coverage consists of two main insurance schemes, a formal mandatory health insurance (MHI), currently run by CNAM, and a state‐subsidized medical assistance scheme (MAS). The MHI covers the public and the private sector employees as well as self‐employed workers. Most of those ineligible for MHI scheme are entitled to MAS, publicly funded and managed by the Ministry of Social Affairs. Individuals living on the poverty line or earning the minimum wage rate are entitled to receive “Free medical cards” or a “reduced‐fee plans” respectively, both accepted by public sector healthcare services.

Data from the Tunisian HealthCare Utilization and Morbidity Survey showed that 66% of the population are covered by MHI, 22% benefit from MAS, while about 12% remains without access to health insurance coverage (National institute of public health 2008). In recent years, a few private insurance schemes were developed but only benefited a small portion of the population (Abu‐Zaineh et al., 2013).

Recent data showed that the Tunisian household spend a lot of money on healthcare, and the average out of pocket payments represented almost 45% of the total health expenditure in 2010 (Chahed & Arfa, 2014). Furthermore, although CNAM partially covers birth‐related health expenses at private facilities, it does not cover costs related to premature births, which are much higher.

Although the health insurance package allows the enrollees to use providers from private and public facilities, and despite the existence of two insurance schemes, the system has its flaws. The reimbursement mechanisms limit coverage for a predetermined list of chronic illnesses and surgical interventions, and reimbursement is subject to an annual expenditure capita per household. Moreover, the public facilities are underfunded; in particular, after 2011, many medications and lab tests are not available, and the number of specialist and doctors is insufficient outside big cities and coastal regions. In addition and because of the limited budget of these facilities and the inefficient management, most of the equipment is not serviced and never replaced. On the other hand, the private sector entails high copayments and extra fees making it impossible for a large number of inhabitants to afford it.

## 5 HEALTH SYSTEM ORGANIZATION

Since 1982, the country implemented a large network of primary health care centers. Currently, health care services are provided by three different sectors: public, private, and parastatal.

Based on the national Statistics Institute and the Ministry of Public Health (MoPH), there are currently 14 general hospitals, 22 Institutes, centers and specialized hospitals mainly located in the large urban cities, 36 regional hospitals providing secondary care, 110 public district hospitals (PDHs), and 2,123 basic health centers. The public health infrastructure is expanding; as such new projects are underway including a second University Hospital in Sfax and another four hospitals or centers planned.

The private sector is the second provider of health care in the country. As of 2014, it operates 82 clinics, with 7,283 medical offices. The parastatal sector is managed by other public departments such as the social security fund (SSF), operates four hospitals, representing 2.5% of hospital beds, six polyclinics, and nine healthcare facilities offering in‐house medical services.

MoPH manages all of the public sector facilities, comprising 85% of total hospital beds and more than 55% of medical personnel (Abu‐Zaineh et al., 2013). Public hospitals receive 55% of total outpatient and inpatient care in the country (Ministry of health. Annual Statistical book of hospital's indicators. Tunisia: Department of publics’ Hospital; 2011).

Despite the increase in the number of hospitals and clinics, about 69% of the total hospital capacity is concentrated in the eastern coastal region of the country. Because of these striking regional differences, many areas have no access to medical services. In 2012, hospital bed density reached 2.1 per 1,000 (Ministry of health, MoH). Overall, the average medical doctor's density is about 1.1 per 1,000, however, it is remarkably uneven, ranging from 3.3 to 3.5 doctors for every 1,000 people in the governorates of Tunis, Sfax and Medinine to 0.2 to 0.4 in the south of the country.

In 2014, the total expenditure of Tunisian healthcare system was about 7% of GDP (gross domestic product). Although it is covering 80% of the population, the public sector receives only 20% of the total health expenditures, while the private facilities benefit from 60%.

## 6 HEALTH SERVICES AND COVERAGE INDICATORS

Just after the declaration of independence in 1956, the “Code of Personal Status” was instituted. These laws were one of the most revolutionary in the Arab and Islamic world. Tunisia was the first country in the region to abolish polygamy. The law also ensured the right to equal pay for men and women. Since 1959, Tunisian woman received the right to vote and to stand for election.

The right to use contraceptives was protected under law since 1961. A family planning program was instituted in 1966, raising the legal age of marriage and providing free access to contraception and counseling for all women throughout the country. Contraceptive usage rate was estimated at 62.5% in 2012 (WHO). First in Africa, this plan also aimed to slow the population growth. The fertility rate is one of the lowest in the region, decreasing from seven children per woman in 1960 to only two currently (CIA world factbook: https://www.cia.gov/library/publications/the-world-factbook/geos/ts.html). The National Board for Family and Population was later created in 1973, and abortion was legalized for any woman within the first trimester regardless of husband's approval (Hajri, Raifman, Gerdts, Baum, & Foster, 2015).

Reproductive health services are provided mainly by a network of 2,091 primary health care centers, 36 reproductive health centers, and 20 youth centers [Ministère de la Santé, Direction de la Santé et de la Planification, *Carte sanitaire 2011* (Tunis: Ministère de la Santé, May 2013)]. However, they are unevenly distributed between the remote rural areas and more industrialized regions. For more detailed information, please refer to the following site: http://www.santetunisie.rns.tn/images/articles/csfinale2011.pdf Since 2001, primary health care services include prevention and treatment of sexually transmitted infections with free and confidential HIV tests; furthermore, antiretroviral drugs are freely provided.

During the last 50 years, Tunisia made big progress in increasing life expectancy and decreasing infant mortality. The average life expectancy was 74.9‐year old in 2014 (73.9 for men and 77.4 for women) with an increase by 12.8 years compared to 1980 (Human development report 2015: http://www.factfish.com/statistic-country/tunisia/human%20development%20index). A decline of infant mortality rate (before reaching 1 year of age) was achieved, decreasing from 178.7 per 1,000 in 1962 to 51.4 per 1,000 in 1985, and to an estimated 12.1 per 1,000 live births currently. Such rates are two times higher in the rural areas than in the urban ones. In 2015, WHO reported a neonatal mortality rate of 8.2 [5.8–11.4] compared to 16.1 [13.5–19.0] in 2002.

Reproductive health is measured by maternal mortality and adolescent birth rates. For every 100,000 live births, 46 women die from pregnancy related causes, this marks a striking improvement compared to 131 per 100,000 in 1990. Deliveries attended by skilled personnel reached 97.6% in 2013, compared to only 76.3% in 1990. Adolescent birth rate is estimated at 4.6 births per 1,000. The current birth rate is 16.4 births per 1,000 while the death rate is 5.7 per 1,000 inhabitants, 2,411 stillbirths were reported (https://knoema.com/atlas/Tunisia/Infant-mortality).

The incidence of tuberculosis is estimated at 35 per 100,000 people in 2014. This number includes new pulmonary, smear positive, and extrapulmonary tuberculosis cases (Knoema data). Unlike other North African countries, Tunisia has no malaria and only a few cases of HIV. Increased vaccinations among youth led to the almost complete eradication of polio, measles, and neonatal tetanus. In recent years, the life expectancy at birth has increased with 3% of deaths being due to communicable diseases while noncommunicable diseases accounted for 72% of deaths. (http://www.borgenmagazine.com/strengthening-tunisias-healthcare-system/).

The population in Tunisia is now aging. With a change in the life style, the burden has shifted from infectious diseases to the emergence of noncommunicable and chronic diseases (hypertension and diabetes) as well as injuries. The top three causes of mortality are ischemic heart disease, cancers and respiratory tract diseases. An increased prevalence of obesity was recorded, with a rate of 27.1% in adults in 2014 (CIA factbook).

Breast cancer and cervical cancer screening have also been introduced into basic health care services as part of the country's National Cancer Control Plans.

[Ministère de la Santé, Direction de Santé, *Plan pour la lutte contre le cancer 2015–2019* (Tunis: Ministère de la Santé, 2015).]

Tunisia counts 12 officially registered transplant centers (http://www.transplant-observatory.org/). The first organ transplantation surgery was performed in 1986 (kidney transplants); since then transplants extended to other organs including cornea, heart, liver, lungs, and pancreas. Tunisia is one of two Arab countries where heart transplants are practiced.

## 7 GENETIC SERVICES

Several centers across the country offer genetic testing and counseling services, however, they are insufficient, do not cover all the regions of country and are far from being comprehensive. The main three genetic clinics are located in the following hospitals located at three coastal cities: Charles Nicolle hospital in the Capital Tunis, Farhat Hached hospital at Sousse, and Habib bourguiba hospital at Sfax.

Very recently, a new unit for congenital and inherited diseases was created at the “Mongi Slim hospital” at Al Marsa where cytogenetic services are also provided. Furthermore, genetic services are also offered as part of research at different centers and at Universities. The main ones are at Pasteur Institute and at the National Neurology Institute. There are few private genetic laboratories with two in Tunis and one in Sfax.

Premarital genetic counseling is mandatory for all couples with family history of genetic diseases and/or consanguinity. This program was implemented in 1964, and then generalized and made mandatory for the entire Tunisian territory in 1995. It was intended to prevent the spread of contagious diseases and limit the frequency of congenital and hereditary diseases in the next generation. It is also an educational program for the future parents, providing information concerning contraception, pregnancy, vaccination, prevention, and counseling. These premarital tests are provided free of charge if done in the public facilities.

Prenatal and postnatal services are available. Fetal ultrasound screening is routinely performed during pregnancy although it is only mandatory during the first trimester. Reliable detection of fetal anomalies relies on how well trained the operator is and how accurate the equipment is. Unfortunately, both criteria are lacking in health facilities in rural areas.

Prenatal diagnosis of chromosomal disorders was available since 1989. Both chorionic villous sampling and amniocentesis are routinely performed upon request and for at‐risk pregnancies. Advanced maternal age is one of the most common reasons for such invasive procedures. It is offered free of charge as a public service, but it is not mandatory and it is up to the couple to decide if they want to get it.

Unfortunately, women living in the more rural and thus poorer areas have less access to prenatal services, monitoring and postnatal follow‐up. According to the WHO 2008 report, “approximately 75% of maternal mortality was due to avoidable causes”. Fetal and neonatal outreach programs should be implemented, especially in less‐favored regions in the country. Tunisia does not have an established national screening program for carriers; such service is mainly done on a research basis.

## 8 GENETIC DISORDERS IN TUNISIA

Genetic disorders constitute a real public health problem and lead to economic and social disadvantages. They constitute a significant burden for the family as well as the healthcare system.

More than 400 genetic disorders were recently assessed in Tunisia. Congenital malformations and chromosomal abnormalities constitute 30% of all genetic disorders found in the Tunisian population, followed by endocrine and metabolic disorders (19%) and nervous system disorders (11%) (Romdhane & Abdelhak, 2011). Because of the high consanguinity rates, an estimated sixfold increase risk of autosomal recessive (AR) diseases was previously reported (Ben Halim et al., 2013). A recent retrospective study including 425 Tunisian patients with AR disorders detected consanguinity in 69.4% of the cases with a prevalence of first cousin marriages of 48.94%. Consanguinity reaches 65.26% in some southern regions. Most of the mutations were found in the homozygous state. Moreover, geographic endogamy was observed in 93.92% of the cases studied. The authors estimated a sevenfold increase in AR diseases associated with consanguinity; this risk was about 24‐fold in certain cases (Ben Halim et al., 2016).

Autosomal dominant disorders represent 23% of all genetic disorders reported in the country, while 5.4% are X‐linked (Romdhane & Abdelhak, 2011). More than one genetic condition per family, referred to as comorbidity, was also recently reported (Romdhane et al., 2016).

A recent study showed that in Tunisian patients, among 174 genetic diseases with identified molecular defect, 73 (41.9%) were founder mutations. For the majority of them, geographic distribution is limited to a region of the country (Romdhane et al., 2012). As an example, the deletion identified in *IL12B* in patients with Mendelian susceptibility to mycobacterial disease (MSMD) is only present in the village of Akouda in central Tunisia (Governorate of Sousse) as shown by haplotype analysis (Ben‐Mustapha et al., 2014; Elloumi‐Zghal et al., 2002).

Identification of founder mutations facilitates the diagnosis of most common diseases at a reduced cost. Besides early diagnosis, it also provides an opportunity for genetic counseling for future pregnancies and thus disease prevention.

The Mediterranean Founder Mutation Database (MFMD: http://mfmd.pasteur.ma/) is a great online resource providing information for 383 founder mutations found in 210 genes related to 219 diseases reported in the Mediterranean population (Charoute et al., 2015). Another resource is “The Catalogue for Transmission Genetics in Arabs “CTGA” database”, which provides a breadth of information about mutations found in a number of Arab countries (Tadmouri, Al Ali, Al‐Haj Ali, & Al Khaja, 2006). Large consanguineous families contributed to the identification of causative loci/genes of a number of AR diseases. Homozygosity mapping in Tunisia was first used to localize Friedreich ataxia with selective vitamin E deficiency locus to chromosome 8q (Ben Hamida et al., 1993). Other studies are listed later in this paper.

## 9 HEMOGLOBINOPATHIES AND OTHER BLOOD DISORDERS

Hemoglobinopathies are the most common genetic disorders in Tunisia, and pose a major public health problem. Carrier frequency was estimated at 4.48%, although it can reach 12.5% in some at‐risk regions. The frequency of β‐thalassemia trait was about 2.21% while the frequency of hemoglobin S carrier was estimated at 1.89% (Fattoum, 2006; Ouali et al., 2016). Similarly, another study reported an average frequency of 1.9% for sickle cell disease in the country (Anwar, Khyatti, & Hemminki, 2014). Thalassemia and sickle cell disease are reported to be epidemic especially in the North West of the country (Haj Khelil et al., 2004, 2010). Such high frequencies were mainly due to the positive selection pressure following the malaria epidemic that ravaged the early farmers. Infected mosquitoes were attracted by the soft and marshy soil leading to an epidemic and the emergence of disorders like sickle cell disease, beta thalassemia, and glucose‐6‐dehydrogenase deficiency (G6PD) (Tadmouri, Sastry, & Chouchane, 2014). Statistics about other hemoglobinopathies in Tunisia can be found on the ITHANET portal (http://www.ithanet.eu/db/ithagenes). ITHANET is a network covering thalassemias as well as other hemoglobinopathies, combining international clinical and research expertise and comprising 26 organizations from 16 countries, including Tunisia (Lederer et al., 2009).

Prenatal diagnosis of hemoglobinopathies was first made available in 1986 at the laboratory of biochemistry and molecular biology at children's hospital of Tunis, but was only fully functional in 1994. Recently, prenatal diagnosis data for 18 years of experience (1994–2012) was reported. Of 461 fetuses of 340 at‐risk couples, 121 were affected. 41% were at risk for beta thalassemia major while 40.3% were at risk for sickle cell disease. A high consanguinity rate among the affected was noted, and a total of 13 β‐thal mutations were identified. The mutation spectrum revealed a great molecular heterogeneity in Tunisia, with more than 28 different alleles reported to date (Chouk et al., 2004; Sahli et al., 2016).

G6PD is the most prevalent enzyme deficiency. It is classified into five classes depending on the residual enzyme activity and clinical severity in accordance with the World Health Organization. A previous survey of the Tunisian population has estimated the incidence of the deficiency at 4%. However, a much higher frequency was found in the central western, northwestern and southeastern high‐risk regions (Guellouz et al., 2010). In Tunisia, the G6PD*A‐ or Matera variant (c.202G>A; p.Val68Met) is the most prevalent among G6PD patients and causes a severe phenotype hemolytic anemia following the ingestion of fava beans. G6PD*Mediterranean (c.563C>T; p.Ser188Phe) and the G6PD*Aures (c.143T>C; p.lle48Thr) mutations were also present in the population (Daoud et al., 2008). GdA‐ and Gd Med constitute 56.8%–89.2% of the molecular spectrum. A recent study found that 67.5% of the deficient population carries the Gd A‐ while only 15% harbor the Md Med allele (Haloui et al., 2016; Laouini et al., 2013). A newborn screening test was carried on 976 neonates from different regions of the country. The frequency of G6PD deficiency was about 4.4%, mirroring that of hemoglobinopathies estimated at 4.48% (Guellouz et al., 2010).

Although known as a rare disorder, Fanconi anemia (FA) occurs at high frequency in Tunisia. The “Tunisian Fanconi Anemia registry (TFAR)” created in 2009 included 142 patients from 118 families diagnosed between 1983 and 2008 of which 86% are consanguineous. Only 19% of these patients received bone marrow transplant. The latter became available in 1999 in one center “Centre tunisien de greffe de moelle osseuse” (Hadiji Mseddi et al., 2012) in Tunis.

Fanconi anemia is genetically heterogeneous with different complementation groups reported. In Tunisia, FA group A is the most encountered with a frequency of 94% (Bouchlaka et al., 2003). A recent study of 83 families, 74 of them are unrelated, including 95 FA patients, revealed the presence of a common haplotype in 42 patients from 26 families and mostly originating from the South of Tunisia. This haplotype was always associated with the deletion of exon 15 in *FANCA*. Another founder mutation in *FANCA* (c.890_893del in exon 10) was identified in Tunisian Jewish patients (Tamary et al., 2000). Identification of founder mutations will provide a rapid and cheap molecular diagnostic tool allowing prenatal testing, genetic counseling, and better bone marrow donor selection. Moreover, an accurate diagnosis is crucial for the choice of therapy; a standard protocol for diagnosis of FA was developed. The chromosomal breakage test allowed the differentiation between mosaic FA patients and non‐FA patients (Talmoudi et al., 2013).

## 10 CONGENITAL MALFORMATIONS

The absence of an established national registry makes it hard to have an accurate estimate of occurrences of birth defects in the country. Epidemiological data are scarce and often regional, as studies have been conducted in maternity wards of main hospitals. Furthermore, underdiagnosis due to the limited access to prenatal care in certain areas together with the lack of awareness and access to genetic testing lead to underestimated statistics. Data from Neonatal screening surveys of congenital malformations in liveborn babies revealed a comparable incidence [3.6% (Chaabouni, Nemsia, Riou, Largueche, & Ferchiou, 1986), 3.7% (Khrouf et al., 1986)] in different regions of the country.

Numerous studies reporting mutations in genes causing congenital malformations have also been reported in recent years; some of the most prevalent are discussed below.

Congenital heart defects represent a public health problem in a developing country like Tunisia. Some of those go under recognized and result in high mortality rates and heavy economic burdens. A retrospective study of 37,294 births registered between 2010 and 2011 revealed an estimated incidence of congenital heart disease of 6.8 per 1,000 births. The most frequent anomaly was ventricular septal defect (VSD), identified in 31% of cases, while coarctation of the aorta and tetralogy of Fallot were found in 4.3% and 6.2%, respectively. About 23% of the patients died and of those 75% occurred before undergoing surgery (Abid et al., 2014). In another recent study of 10,447 newborn cases, the prevalence was estimated at 2.77 per 1,000 live births (Methlouthi et al., 2016). A multicenter study is needed to establish an accurate rate.

Neural tube defects are another group of major congenital anomalies with a prevalence of 2.02/10,000 live births in the country. Over a period of 20 years (1991–2011), 769 stillborns with neural tube defects were recorded; 59.5% of the cases were females. Spina bifida was the most frequent (38.9%), followed by anencephaly (22.8%) and encephalocele (17.8%). The consanguinity rate was estimated at 36.7% (Nasri et al., 2014). It should be noted that fortification of food products with folate is not mandatory for pregnant women in Tunisia.

Disorders of sex development (DSD) are also prevalent in Tunisia similar to other populations with high incidence of endogamy. Congenital adrenal hyperplasia (CAH) due to 21‐OH hydroxylase deficiency is particularly prevalent; screening of the *CYP21B* gene revealed that the most common pathogenic variant in the Tunisian patients is p.Q318X (Kharrat et al., 2004). Molecular analysis of 15 unrelated patients suffering from 11β‐hydroxylase deficiency revealed two homozygous pathogenic variants within the *CYP11B1* gene: p.Q356X and p.G379V. The latter had a prevalence of 73.3% among affected patients (Kharrat et al., 2010). More recently, a study of patients with 17β‐HSD3 deficiency, a rare recessive form of 46,XY DSD, revealed genetic heterogeneity. A high carrier frequency of the p.C206X pathogenic variant within the *HSD17B3* gene was found at 1 in 40. All carriers harbored the same haplotype suggestive of a founder effect (Ben Rhouma et al., 2017).

Bardet‐Biedl syndrome (BBS) is relatively rare, with an estimated prevalence of 1 in 156,000 (M'hamdi, Ouertani, Maazoul, & Chaabouni, 2011). Mutations were frequently found in *BBS1* (37%) and *BBS2* (18%) (M'hamdi et al., 2014), and founder mutations identified in both *BBS2* and *BBS8* (p.R189X and c.459 + 1G>A, respectively) (Smaoui et al., 2006).

## 11 CHROMOSOMAL ABNORMALITIES

Increased risk for chromosomal abnormalities is due in part to limited access to family planning, diagnosis and associated services, in addition to childbearing at older maternal ages, teenage marriages in the rural areas, and deficient or absent prenatal screening. Consanguinity does not seem to affect the frequency of aneuploidies in the population. Since 1980, karyotyping is the first line of cytogentic analysis in the country. Currently, in situ hybridization as well as array CGH are available for the detection of constitutional abnormalities. Prenatal diagnosis of 3,110 fetuses revealed an incidence of 4.18% of abnormal karyotypes. Among those, 72% were aneuploidies and 40% were trisomy 21 cases (Chaabouni et al., 2001). Today pregnant women are offered noninvasive prenatal testing (NIPT) in the private sector to avoid amniocentesis and chorionic villus sampling (CVS).

The prevalence of Down syndrome in Tunisia is estimated at 0.98 per 1,000 pregnancies (Chelli et al., 2008). Analysis of 248 patients with cytogenetically confirmed Down syndrome revealed that 22% of patients are the product of consanguineous marriages, mirroring the consanguinity rate (Kelmemi, Chelly, Kharrat, & Chaabouni, 2015).

Cases of Turner syndrome (TS) were also reported in Tunisia. A 21‐year retrospective study showed that 24% of patients with Turner syndrome were diagnosed in adulthood (Elleuch et al., 2010) compared to 48% in another study of 89 cases of TS (Kammoun et al., 2008). Congenital heart defects were noted in 50% of the patients and autoimmune diseases in 39.1%. Dysmorphic features were observed in 85% of cases, mostly in monosomic patients (45,X) (Elleuch et al., 2010).

The occurrence of chromosomal abnormalities in patients with nonobstructive azoospermia and severe oligo‐asthenospermia was estimated at 21.7% and 13.5%, respectively (Ghorbel et al., 2012). In a recent study of 476 infertile men, chromosomal abnormalities were identified in about 10.9% of cases. Similarly, abnormal findings were higher in the azoospermia group compared to the severe oligozoospermia group (14.02% and 4.05%, respectively) (Amouri et al., 2014).

Several cases with chromosomal disorders are reported in the literature, including aneuploidy, chromosomal rearrangements and microdeletion syndromes (Abdallah‐Bouhjar et al., 2013; Bouhjar et al., 2012). A recent study of 38 cases of Williams‐Beuren syndrome revealed the presence of dysmorphic features in all patients and cardiovascular anomalies in 66%; while about 8% presented with normal intelligence (Ouertani et al., 2014).

## 12 DEAFNESS

The high rate of consanguinity contributed to the preponderance of deafness in Tunisia, with a high prevalence of nonsyndromic hearing loss ranging between 2% and 8% in some of the isolates in the northern villages (Ben Arab et al., 2004). The AR forms (DFNB) are the most frequent of the prelingual genetic forms representing 80% of the studied cases. Assortative mating resulted in the coexistence of different etiologies of hearing loss disorders along with aminoglycoside ototoxicity (Ben Arab et al., 2004).

The high frequency of sensorineural deafness elicited research groups in the country to collect families and to identify the genetic etiology of their deafness in collaboration with other North African and European groups. Several new loci were thus first identified in Tunisian families (Table 1), and many mutations were detected. One of the first loci was DFNB1 (*GJB2*), mapped to Chr13q11‐q12 by linkage analysis performed on two large consanguineous Tunisian families exhibiting AR hearing loss (Guilford et al., 1994).

**Table 1:** Deafness genes identified in Tunisian families

Disease	Locus	References
ARNSHL DFNB1	Locus mapped to 13q	Guilford et al. (1994)
DFNB2	*MYO7A*	Weil et al. (1997)
Pendred syndrome	*SLC26A4*	Masmoudi, Charfedine, et al. (2000) Charfeddine et al. (2010)
ARNSHL DFNB10	*TMPRSS3*	Masmoudi et al. (2001)
Thiamine responsive megaloblastic anemia syndrome with diabetes and deafness	*SLC19A2*	Gritli et al. (2001)
ARNSHL DFNB32	1q13.3‐22.1	Masmoudi et al. (2003)
USH1G	*SANS*	Weil et al. (2003) Chakchouk, Ben Said, et al. (2015), Chakchouk, Grati, et al. (2015) Riahi et al. (2015)
ARNSHL DFNB13	Refined the locus to a 2.2 Mb	Masmoudi et al. (2004)
ARNSHL DFNB66	Locus mapped to 6q21.2‐22.3	Tlili, Männikkö, et al. (2005)
ARNSHL DFNB31	*WHRN*	Tlili, Charfedine, et al. (2005)
Brittle cornea syndrome	Locus mapped on 16q24	Abu et al. (2006)
ARNSHL DFNB63	11q13.3‐q13.4	Tlili et al. (2007)
AD deafness DFNA36	*TMC1*	Tlili et al. (2008)
ARNSHL DFNB7/B11	*TMC1*	Tlili et al. (2008) Ben Said et al. (2010) Riahi et al. (2014)
USH2	New locus on 15q	Ben Rebeh, Benzina, et al. (2008)
USH1B	*MYO7A*	Ben Rebeh, Morinière, et al. (2008) Riahi et al. (2015) Ben‐Rebeh et al. (2016)
ARNSHL DFNB63	*LRTOMT*	Ahmed et al. (2016) Riahi et al. (2014)
ARNSHL DFNB1A	*GJB2*	Alemanno et al. (2009) Riahi, Hammami, et al. (2013), Riahi, Zainine, et al. (2013) Trabelsi et al. (2013)
AR retinitis Pigmentosa (ARRP)	*PDE6B*	Hmani‐Aifa et al. (2009)
ARNSHL DFNB3	*MYO15A*	Belguith et al. (2009) Riahi et al. (2014)
Usher syndrome type 2A	*GPR98*	Hmani‐Aifa et al. (2009)
ARNSHL DFNB80	Locus mapped to 2p16.1‐p21	Mosrati et al. (2013)
AD DFNA3	*GJB2*	Riahi, Hammami, et al. (2013), Riahi, Zainine, et al. (2013)
USH1C	*USH1C*	Chakchouk, Ben Said, et al. (2015) Ben‐Rebeh et al. (2016)
ARNSHL DFNB53	*COL11A2*	Chakchouk, Grati, et al. (2015)
ARNSHL DFNB66	*DCDC2a*	Grati et al. (2015)
ARNSHL DFNB60	*SLC22A4*	Ben Said et al. (2016)
USH1F	*PCDH15*	Ben‐Rebeh et al. (2016)

ARNSHL, autosomal recessive nonsyndromic hearing loss.

*GJB2* is the gene most frequently involved in AR deafness in Tunisia; causative variants were identified in 39% of families with congenital hearing loss. The most common pathogenic variant was c.35delG, accounting for 35% of the cases and 85.4% of all *GJB2* mutant alleles identified (Riahi, Hammami, et al., 2013). The carrier frequency of c.35delG in the general population in Tunisia was reported to be 1.3% (Masmoudi, Elgaied‐Boulila, et al., 2000).

Fifty‐eight mutations in 21 different genes have been reported in North African families with hearing loss, all included in the North African DNA Mutation Database (http://www.tdvd.org/northafrica/affiche.php?alpha=D). The “North African Deafness Chip”, a diagnostic oligonucleotide array using multiplex‐PCR coupled with dual‐color arrayed primer extension and targeting these 58 mutations was developed, allowing rapid, accurate, and cost effective method to screen for common mutations in the region (Chakchouk, Ben Said, et al., 2015, Chakchouk, Grati, et al., 2015).

Usher syndrome, another clinically and genetically heterogeneous AR disorder is also responsible for deafness although it is underdiagnosed in Tunisia (Adato et al., 1997). An extensive ophthalmological examination is needed to detect retinitis pigmentosa and peripheral alterations of the retina in patients with congenital profound deafness. This depends on the availability of electroretinography and OCT imaging which is unfortunately limited to tertiary health centers. This hampers the early diagnosis of Usher syndrome, where cochlear implant in early childhood was proven to be beneficial. Identification of the etiology of hearing loss in young children allows early intervention leading to a better linguistic development, social and cognitive skills.

## 13 METABOLIC DISORDERS

A metabolic clinic was instituted in 1987 at “La Rabta Hospital” in the capital. The organization of metabolic schools since 1988 further contributed to improved awareness of physicians about inborn metabolic disorders.

A number of studies reporting mutations in genes causing Inborn errors of metabolism have been published (Table 2). Among those, some are believed to be founder mutations, information that is helpful for future premarital counseling and carrier screening (Khedhiri et al., 2012). However, metabolic disorders remain under diagnosed in Tunisia. Most of the enzymatic tests are not offered in the country and are sent to European laboratories.

**Table 2:** Reported mutations in some metabolic disorders encountered in Tunisia

Disease	Gene	Mutations	References
Maple syrup disease (MSUD)	*BCKDHB* *DBT*	p.Glu239Gly c.1333_1336delAATG (aka p.Asn445Ter)	Jaafar et al. (2013)
PKU	*PAH*	p.Leu48Ser p.Ser349Pro	Bercovich et al. (2008) Weinstein et al. (1993)
Cystinosis	*CTNS*	Absence of 57Kb del p.Gly308Arg c.771_793del23 Ex1‐Ex5del; 20Kb del	Chkioua et al. (2015)
MPS I Hurler Hurler‐Scheie	*IDUA*	c.1805delTinsGAACA (aka p.Phe602Ter) p.Ile270Ser p.Arg628Ter p.Pro533Arg p.Tyr581Ter p.Leu578Gln p.Phe177Ser c.1587_1588insGC (aka p.Leu530ArgfsX31)	Laradi et al. (2005) Chkioua et al. (2007) Chkioua, Khedhiri, Kassab, et al. (2011) Chkioua, Khedhiri, Ben Turkia, et al. (2011)
MPS II Hunter	*IDS*	p.Arg88Pro	Chkioua, Khedhiri, Ferchichi, et al. (2011)
Sanfilippo Syndrome MPSIIIA	*SGSH*	p.Met1? p.Ser66Trp p.Arg377Cys p.Gln365Ter c.29dupC (p.Leu11AlafsX22) c.1080delC (aka p.Val361SerfsX52) Ex1‐Ex5del	Ouesleti et al. (2011)
MPSIIIB	*NAGLU*	p.Tyr558Ter p.Pro604Leu (Founder)	Ouesleti et al. (2011)
MPSIIIC	*HGSNAT*	p.Trp403Ter (Founder) p.Tyr627Cys (Vus)	Ouesleti et al. (2011)
MPS IV Morquio A	*GALNS*	c.120 + 1G>A p.Gly66Arg p.Ala85Thr p.Asp288Gly p.Arg386Cys	Khedhiri et al. (2007) Khedhiri et al. (2009) Khedhiri, Chkioua, Ferchichi, Miled, and Laradi (2011) Khedhiri et al. (2012) Chkioua et al. (2016)
Gaucher disease	*GBA*	p.Asn370Ser p.Leu444Pro (aka Leu483Pro) RecNciI	Cherif et al. (2009)
Metachromatic leukodystrophy	*ARSA*	p.Ile179Ser (aka p.Ile181Ser)	Barboura et al. (2011)
Primary hyperoxaluria	*AGXT*	p.Pro11Leu (DFP) p.Pro28Ser p.Gly41Arg p.Ile56Asn p.Gly82Arg p.Gly190Arg p.Gly156Arg p.Trp108Arg p.Ser205Leu p.Ile244Thr p.Arg360Gln c.33dupC (aka p.Lys12GlnfsX156) c.406_410dupCTGCA aka p.Gln137HisfsX19)	M'Dimegh et al. (2016) M'Dimegh et al. (2017) Kanoun et al. (2017)
Tyrosinemia type I	*FAH*	c.554‐1G>T aka IVS6‐1G>T (hot spot)	Nasrallah et al. (2015)
Tysosinemia type II	*TAT*	p.Cys151Tyr p.Leu273Pro c.869dupG (p.Trp291LeufsX6)	Charfeddine et al. (2006) Bouyacoub et al. (2013)

Maple syrup urine disease (MSUD) is one of the most common disorders of amino acid metabolism in Tunisia, with a reported incidence of 1 in 13,716 live births (Hadj‐Taieb et al., 2012). Founder mutations were identified in families originating from central Tunisia (Jaafar et al., 2013; Nagara et al., 2014).

The frequency of phenylketonuria (PKU) was estimated at 1.32% based on screening of mentally retarded patients originating from different centers throughout the country (Khemir et al., 2009). The birth prevalence of congenital hypothyroidism was about 1 in 5,000 based on the data collected between 1996 and 1997 (Chaabouni‐Bouhamed, 2008). The incidence of Hereditary Tyrosinemia type 1 is relatively high in Tunisia, and was estimated at 1/14,804 births with a predominance of the chronic form (Nasrallah et al., 2015).

Based on a retrospective epidemiological survey of biochemically confirmed cases of Mucopolysaccharidoses (MPS) in Tunisia, collected over a period of 35 years, the incidence was estimated at 1 in 44,000 live births. Consanguinity was found in 83% of the affected families. MPS I and III were the most frequent among the affected individuals (Ben Turkia et al., 2009). In the center and south of Tunisia, the incidence of MPS IVA (Morquio) is estimated at 2.8 in 100,000 live births (Laradi et al., 2001).

Gaucher disease (GD) is rare in Tunisia and its frequency is estimated at 1:108,000 live births (Dandana, Ben Khelifa, Chahed, Miled, & Ferchichi, 2016). A study of 30 affected individuals with GD revealed that N370S, L444P and RecNciI mutations account for 74% of the total GD alleles identified in Tunisian patients (Cherif et al., 2009; Table 2). N370S is the most frequent mutation among Tunisian patients (53%) similar to southern European countries (Ben Rhouma et al., 2012). This finding suggests that the N370S mutation has been likely introduced in North Africa during the Roman Empire. It is mainly seen at in a heterozygous state in combination with another mutated allele in trans. The L444P was seen in 16% whereas the complex allele RecNciI was detected in 14% alleles (Cherif et al., 2009).

Primary hyperoxaluria type 1 (PH1) is another rare AR disease that is particularly frequent in Tunisia. A high number of patients were diagnosed as adults (48%). A recent study of 146 patients from 135 unrelated families with PH1 and originating from 20 different regions identified consanguinity in 56.8% of the patients. The Maghrebian mutation p.I244T was the most frequently identified (43.4%), and is considered the first cause of PH1 in the country (M'Dimegh et al., 2017). This mutation is associated with individuals of Spanish descent and could have an Iberomaurusian origin. In fact, p.I244T is considered a founder mutation in the Canary Islands with a frequency of 91.6% (Benhaj Mbarek et al., 2011). Molecular diagnosis guided the choice of treatment such as pyridoxine as well as the transplantation protocol.

The first molecular diagnosis of nephropathic cystinosis was performed in 2015. Three patients from three unrelated consanguineous families were tested. The common 57 Kb deletion was not present, but two previously reported variants (G308R, c.771_793del) were identified as well as a novel deletion of 20,327 bp. One of the families benefited from prenatal diagnosis and one patient received cysteamine treatment since diagnosis (Chkioua et al., 2015).

The incidence of CF is unknown and reported to be low, but no epidemiologic study has been done since 2005. This study included 390 affected children from 383 unrelated families recruited from different parts of the country. F508del was the most common mutation (50.74%), followed by G542X (7.96%), W1282X (6.66%) and N1303K (5.92%) (Messaoud et al., 2005) (Table 3). A recent multicenter retrospective study including 33 confirmed CF patients enrolled between 1997 and 2012 was reported. Consanguinity was identified in 47% of these cases, with a higher prevalence of males (70%) and an average age of onset estimated at 6 months. Genetic testing was done in 88% of the patients; 51% of whom carry delta 508 mutation (Halioui‐Louhaichi et al., 2015). The first case of prenatal diagnosis was performed in 1994 in the laboratory of biochemistry of Children's Hospital in Tunis. A 10‐year experience in prenatal diagnosis of CF in Tunisia was recently published. A panel of 17 most frequent mutations in our population was used, with 90% detection rate. Analyzed samples were either chorionic villus (CVS) or amniotic fluid; maternal cell contamination was concurrently tested using a set of polymorphic microsatellite markers. Similar to previous reports, these data showed that F508del is the most common mutation (51.28%), followed by E1104X (12.82%), N1303K and G542X (8.97%). All the other mutations were detected in less than 7% of the tested individuals (Hadj Fredj et al., 2015). No premarital carrier screening is offered for cystic fibrosis, and prenatal diagnosis is mainly restricted to the capital and the big coastal cities. No prevention measures are put in place and therapeutic options are very limited and expensive.

**Table 3:** Most common *CFTR* mutations detected in Tunisia

Mutations	References
p.Trp19Ter p.Arg74Trp p.Gly85Glu p.Tyr122Ter p.Ile148Thr p.Val201Met p.Gly542Ter (Phoenician) p.Thr665Ser p.Val754Met p.Arg785Ter p.Leu1043Arg p.Arg1066Cys p.Glu1104Ter p.Arg1158Ter p.Phe1166Cys p.Ile1203Val p.Asp1270Asn (Vus) p.Trp1282Ter p.Asn1303Lys c.273 + 1G>A aka c.405 + 1G>A c.579 + 1G>T aka c.711 + 1G>T c.1521_1523delCTT aka F508del c.1679 + 5A>G aka c.1811 + 5A>G c.2637_2644del8 aka c.2766del8 c.3598delAinsTCT aka c.3729delAinsTCT c.3718‐2477C>T aka c.3849 + 10KbC>T c.3889dupT aka c.4016insT c.4136 + 2T>G aka c.4268 + 2T>G	Kerem and Kerem (1995) Quint, Lerer, Sagi, and Abeliovich (2005) Messaoud et al. (2005) Fredj et al. (2009) Boudaya, Fredj, Siala, Bibi, and Messaoud (2013) Hadj Fredj et al. (2013) Hadj Fredj et al. (2015) Oueslati et al. (2015)

Although newborn screening is not yet established, Tunisia has pilot screening programs that test a portion of the population with the goal to expand to cover all babies.

## 14 NEUROGENETICS

The late professor Mongi Ben Hamida is considered the father of Neurology not only in Tunisia but also in the Maghreb. He founded the National Institute of Neurology in 1973, now the reference center in the North of Tunisia. He was the pioneer in the field of hereditary neuromuscular disorders. The department of neurology at the Institute was the first in Tunisia and North Africa to research the epidemiologic profiles of various neurological diseases and to develop research tools to study some of the most frequently encountered disorders in the country. By establishing an international research collaboration network, his team contributed to mapping and identification of the genetic etiology of a number of neuromuscular diseases.

In 1980, professor Ben Hamida described a new type of severe recessively inherited childhood muscular dystrophy, affecting both males and females in 31 cases from 13 families (Ben Hamida & Marrakchi, 1980). He named it “Tunisian severe childhood AR muscular dystrophy” (SCARMD), now referred to as limb girdle muscular dystrophy type 2C (LGMD2C). In 1992, the locus was mapped to Chr13q12 in three highly inbred Tunisian families (Ben Othmane et al., 1992). The gene encoding for gamma‐sarcoglycan subunit was later identified, and one homozygous deletion c.521delT was found in all tested patients, indicating a founder effect (Noguchi et al., 1995). Although LGMD2C is the most common form in the region, mutations in the other subunits were also identified. The first Tunisian family with beta‐sarcoglycanopathy (LGMD2E) was reported in 1998, a homozygous variant p.R91L was identified (Bönnemann et al., 1998). Furthermore, a novel locus for AR limb girdle muscular dystrophy LGMD2I was identified in a large consanguineous Tunisian family, with 13 affected individuals, mapping to chr19q13.3 (Driss et al., 2000).

Professor Ben Hamida described eight patients belonging to two large inbred families with selective vitamin E deficiency and a Friedreich ataxia phenotype. Linkage analysis and homozygosity mapping were later performed on a total of three families including 12 affected individuals. Friedreich ataxia with vitamin E deficiency (AVED) locus was thus identified and mapped to Chr8q (Ben Hamida et al., 1993). A recent study of a large cohort of 131 Tunisian patients (48 families) showed that 91.7% of the 77 patients molecularly tested were homozygous for the c.744delA mutation in *TTPA* gene, suggesting a founder effect. All patients received vitamin E supplementation as a treatment (El Euch‐Fayache, Bouhlal, Amouri, Feki, & Hentati, 2014).

Linkage analysis in a large Tunisian family comprising several members presenting with an AR cerebellar ataxia allowed the mapping of gene locus to chr13q11‐12 (Mrissa et al., 2000). The same research group mapped a recessive form of familial amyotrophic lateral sclerosis to chr2q33‐q35 in 10 affected members of an inbred ALS2 Tunisian family (Hentati et al., 1994).

The first large Tunisian kindred with Giant axonal neuropathy (GAN) was described in 1990. All patients had a relatively slow course of the disease (Ben Hamida, Hentati, & Ben Hamida, 1990). Subsequently, linkage and haplotype analysis in this family together with two other consanguineous families led to mapping of *GAN1* to chr16q24.1 (Ben Hamida et al., 1997). This region was further narrowed to a less than 590 KB critical interval (Cavalier et al., 2000). The *GAN* gene was later identified in collaboration with a French group, confirming the power of homozygosity mapping in such consanguineous families (Bomont et al., 2000).

Linkage analysis of a large Tunisian family with 13 out of the 19 affected members presenting with severe axonal AR form of Charcot‐Marie‐Tooth disease with pyramidal features, led to the identification of a new locus CMT4C2 on Chr8q21.3 (Barhoumi et al., 2001). No responsible gene has been identified yet.

Another locus CMT4A on chr8q13‐q21 was identified in Tunisian inbred families with early age of onset and severe clinical phenotype. Patients presented with an AR demyelinating peripheral neuropathy (Ben Othmane et al., 1993). Further positional cloning allowed the identification of *GDAP1* gene, encoding ganglioside‐induced differentiation‐associated protein‐1, as the causative gene in four different Tunisian families. Three different mutations were detected showing allelic heterogeneity in the region (Baxter et al., 2002).

Using homozygosity mapping in a large six generation Tunisian family including six individuals with an AR demyelinating hereditary motor and sensory neuropathy, a locus for the AR CMT4B2 with folding myelin sheaths was mapped to chr11p15 (Ben Othmane et al., 1999). Later, linkage analysis of one Tunisian family with four affected members and one Moroccan family with three affected members allowed the identification of a candidate interval overlapping with the previously mapped CMT4B2 locus. Mutations within the *SBF2* (aka *MTMR13*) were identified (Azzedine et al., 2003).

In a recent report, a cohort of 100 CMT patients belonging to 57 families collected over 12 years was molecularly analyzed. Among the patients with predominance of demyelinating forms, *PMP22* gene duplication was the most frequent mutation (61.1%) followed by *GDAP1* (22.2%) and *MPZ* gene (11.1%) (Nasri et al., 2015).

Genome‐wide screening (6090 SNP markers) of the members of a consanguineous Tunisian family (five affected and eight unaffected) with a complicated HSP‐thin corpus callosum led to the identification of a new locus SPG46 on chromosome 9 (Boukhris et al., 2010). Later, mutations in *GBA2* were identified (Hammer et al., 2013; Martin et al., 2013).

Linkage analysis conducted on eight families where three were from Tunisia allowed the refinement of the SPG15 locus to a 2.64 Mb region as well as the identification of *ZFYVE26* as the causative gene (Boukhris et al., 2008; Hanein et al., 2008). The minimal prevalence of HSP in the governorate of Sfax was reported to be 5.75/100,000 inhabitants; recessive inheritance was reported in 97.3% of the patients in the region with the high prevalence of complex forms. *SPG11* and *SPG15* constitute the major mutated loci in Tunisia (Boukhris et al., 2009).

Familial Parkinsonism is due to mutations within the Leucine—rich repeat kinase 2 (*LRRK2*) gene. The variant p.Gly2019Ser was found to confer a high genotypic and attributable risk (Hulihan et al., 2008). Up to 30% of patients with sporadic Parkinson's disease carry this variant in Tunisia and 30%–40% in the North African Arab‐Berber population compared to 13% in Ashkenazi Jews and 1% in caucasian populations (Lesage et al., 2006; Trinh et al., 2014; Warren et al., 2008). Incomplete penetrance and variable age of onset were reported. Recently, using genome‐wide linkage analysis on 41 multi‐incident Arab‐Berber families including 253 carriers, followed by locus‐specific association analyses, *DNM3* was identified as an age of onset modifier of the disease (Trinh et al., 2016).

There is no formal screening for learning disabilities in Tunisian schools. The undiagnosed prevalence rate of attention deficit disorder (ADHD) reached 10% in the region of Sfax for students between the ages of 6 and 12. Among all the students between the ages of 7 and 9, 8% had undiagnosed learning disabilities (Shaub, 2016).

A study of 200 Tunisian boys with mental retardation showed a frequency of 7.6% for Fragile X syndrome cases (Ben Jemaa et al., 2008). The prevalence of Fragile X is much higher in the Tunisian Jews due to a founder effect of a rare haplotype consisting of *FMR1* CGG repeats completely devoid of AGG interruptions (Falik‐Zaccai et al., 1997; Toledano‐Alhadef et al., 2001).

## 15 XERODERMA PIGMENTOSUM AND OTHER SKIN DISORDERS

Although a rare disease, many cases with Xeroderma Pigmentosum (XP) were reported in the Tunisian population due to the high rate of consanguineous marriages. The frequency of the disease is estimated to be 1 in 10,000. XP is classified into three clinical forms: severe, intermediate and moderate (Zghal, Fazaa, & Kamoun, 2006; Zghal et al., 2003). About one‐third of cases are suspected to be XP‐A. XP is associated with an extreme cutaneous photosensitivity, early onset of persistent erythema, DNA repair defects and a high predisposition to cancers. Some patients develop neurological symptoms (Messaoud, Ben Rekaya, Kefi, et al., 2010). Molecular diagnosis of XP was introduced in 2007; founder mutations were reported among Tunisian XP patients (Ben Rekaya et al., 2009; Messaoud, Ben Rekaya, Cherif, et al., 2010). Of 117 XPA alleles harboring p.R228X described so far, 83 were identified in Tunisian patients (Messaoud et al., 2012) (Table 4).

**Table 4:** Mutations in XP and other skin disorders

Disease	Gene	Mutations	References
XP‐A	*XPA*	p.Glu111Ter p.Arg207Ter p.Arg228Ter c.721dupG	Messaoud, Ben Rekaya, et al. (2013) Messaoud, Ben Rekaya, Kefi, et al. (2010) Messaoud et al. (2012)
XP‐C	*XPC*	c.1643_1644delTG (aka p.Val548AlafsX25) c.779 + 1G>A p.Glu284Ter	Ben Rekaya et al. (2009) Messaoud, Laroussi, et al. (2013) Ben Rekaya et al. (2013) Jerbi et al. (2016)
XP‐V	*POLH*	Ex10 del c.1568_1571delGTCA (aka p.Ser523LysfsX16) c.660 + 1G>A	Ben Rekaya et al. (2014) Ben Rekaya et al. (2011) Messaoud, Laroussi, et al. (2013)
Bloom syndrome	*BLM*	c.1985_1986delAA (aka p.Lys662IlefsX5) c.3620_3621delAA (aka p.Lys1207SerfsX9)	Ben Salah, Salem, Masmoudi, Ben Rhouma, et al. (2014) Ben Salah, Salem, Masmoudi, Kallabi, et al. (2014)
Ataxia telangiectasia	*ATM*	p.Arg35Ter (founder)	Gilad et al. (1996)

Screening of patients with a severe presentation of XP‐C showed genetic homogeneity. One single mutation c.1643_1644delTG was identified, and haplotype analysis suggested a founder effect (Ben Rekaya et al., 2009). Moreover, molecular investigation of 64 XP‐C patients belonging to 44 families revealed the presence of the c.1643_1644delTG mutation in 60 patients (94%) (Jerbi et al., 2016). A map of the distribution of XP‐C in Tunisia is available; please refer to Jerbi et al., 2016;. Some clusters of heterogeneity of the disease were recently reported, with the presence of a second founder mutation within exon 7 restricted to the south of the country (Ben Rekaya et al., 2013).

Sixteen patients belonging to 10 unrelated families diagnosed with XP‐variant were all found to carry a deletion of exon 10; breakpoints were located within homologous Alu sequences. Screening for this common mutation can be simply done by PCR amplification making molecular testing fast and inexpensive (Ben Rekaya et al., 2014).

Recently, whole exome sequencing and Run Of Homozygosity (ROH) analysis were performed for two Tunisian XP patients. This study identified the fisrt cases of complementation groups XP‐D and XP‐E in Tunisia and in North Africa. Pathogenic variants within the *ERCC2* and *DDB2* genes segregated in all affected family members (Ben Rekaya et al., 2017).

Xeroderma Pigmentosum strongly predisposes affected individuals to skin cancers including melanoma in areas exposed to sunlight. The incidence of cutaneous melanoma in Tunisia is relatively low and estimated at 0.5–0.7 per 100,000 inhabitants (Naouali et al., 2017).

The Northern Tunisian cancer registry consists of four editions that cover a period of 13 years (from 1994 to 2006). The Southern Tunisian cancer registry consists of three editions that cover a period of 6 years (1997–2002).

Since this genodermatosis is relatively common in Tunisia with no available therapy, molecular testing and prenatal diagnosis are very valuable at limiting its occurrence in consanguineous families. Such testing is offered but because of its high cost, only a small number of individuals can benefit from it (Messaoud, Ben Rekaya, et al., 2013). A patient support group “Helping Xeroderma Pigmentosum children” was established, providing patients with a better ultraviolet protection (http://www.xp-tunisie.org.tn).

Bloom syndrome, another rare autosomal recessive disorder, is relatively frequent in Tunisia. Sister chromatid exchange (SCE) analysis is used to confirm the diagnosis (Ben Salah, Salem, Masmoudi, Ben Rhouma, et al., 2014, Ben Salah, Salem, Masmoudi, Kallabi, et al., 2014).

## 16 BREAST AND OVARIAN CANCERS, COLORECTAL CANCER

The prevalence of breast cancer is between 16% and 38%. Many patients are referred to Salah Azaiz Cancer Institute, where several epidemiological studies have been conducted. The first molecular study of the role of *BRCA1* in hereditary breast cancer identified five different frameshift variants in 5 out of 32 unrelated patients (Troudi et al., 2008). A splice site variant specific to the Tunisian population (c.212 + 2insG) was identified in *BRCA1* together with a founder North African mutation c.798_799delTT (Mahfoudh et al., 2012). Large rearrangements account for 4%–28% of all inherited *BRCA1/2* mutations. Exon 5 deletion and exon 20 duplication within *BRCA1* gene were identified in Tunisian patients by means of MLPA analysis (Riahi, Chabouni‐Bouhamed, & Kharrat, 2017).

Colorectal cancer has an incidence of 7.4 new cases per 100,000 individuals per year. Cases can be either sporadic or more rarely familial. A large deletion involving exon 6 of *MLH1*, a DNA mismatch repair gene, was observed in a family with six patients diagnosed with a colorectal or an endometrial cancer and characterized by a severe phenotype and an early onset (Aissi‐Ben Moussa et al., 2009; Chouchane & Hassen, 2010).

## 17 CONCLUSION/FUTURE

There is an urgent need to increase genetic literacy among the health care personnel, in particular primary care practitioners, by providing the appropriate education and training. The lack of public awareness toward the early recognition and prevention of inherited diseases is another hurdle. The public must be informed and educated to seek services and counseling by creating support groups, organizing workshops, and by creating a network between the different care providers, the school or institution and the parents.

## 18 USEFUL SITES

http://www.nationsonline.org/oneworld/tunisia.htm
http://www.africa.upenn.edu/Country_Specific/Tunisia.html
http://www.tunisie.online.fr/
http://www.indexmundi.com/tunisia/#Demographics
https://www.unicef.org/infobycountry/Tunisia_statistics.html
http://en.unesco.org/countries/tunisia
https://data.worldbank.org/country/Tunisia
Washington report on Middle East affairs, Nov/Dec 1996: http://www.wrmea.org/1996-November-December/tunisia-in-history-the-country-which-gave-its-name-to-a-continent.html
https://fr.wikipedia.org/wiki/Mus%C3%A9e_national_du_Bardo_(Tunisie)
Health and Human rights journal, 12 September 2016: https://www.hhrjournal.org/2016/09/reproductive-health-policy-in-tunisia-womens-right-to-reproductive-health-and-gender-empowerment/
https://www.oxfordbusinessgroup.com/overview/annual-check-solid-foundation-sector-ready-overhaul

## CONFLICT OF INTEREST

None declared.

## References

[mgg3392-bib-0001] Abdallah‐Bouhjar, I. B. , Mougou‐Zerelli, S. , Hannachi, H. , Gmidène, A. , Labalme, A. , Soyah, N. , … Elghezal, H. (2013). Molecular cytogenetic and phenotypic characterization of ring chromosome 13 in three unrelated patients. Journal of Pediatric Genetics, 2(3), 147–155. 27625853 10.3233/PGE-13063 PMC5020974

[mgg3392-bib-0002] Abid, D. , Elloumi, A. , Abid, L. , Mallek, S. , Aloulou, H. , Chabchoub, I. , … Kammoun, S. (2014). Congenital heart disease in 37,294 births in Tunisia: Birth prevalence and mortality rate. Cardiology in the Young, 24, 866–871. https://doi.org/10.1017/S1047951113001194 24103727 10.1017/S1047951113001194

[mgg3392-bib-0003] Abu, A. , Frydman, M. , Marek, D. , Pras, E. , Stolovitch, C. , Aviram‐Goldring, A. , … Pras, E. (2006). Mapping of a gene causing brittle cornea syndrome in Tunisian Jews to 16q24. Investigative Ophthalmology & Visual Science, 47(12), 5283–5287. https://doi.org/10.1167/iovs.06-0206 17122114 10.1167/iovs.06-0206

[mgg3392-bib-0004] Abu‐Zaineh, M. , Romdhane, H. B. , Ventelou, B. , Moatti, J. P. , & Chokri, A. (2013). Appraising financial protection in health: The case of Tunisia. International Journal of Health Care Finance and Economics, 13(1), 73–93. https://doi.org/10.1007/s10754-013-9123-8 23381233 10.1007/s10754-013-9123-8

[mgg3392-bib-0005] Achour, N . (2011). Le systeme de santé tunisien: Etat des lieux et defis. http://www.unfpa-tunisie.org. (Article in French)

[mgg3392-bib-0006] Adato, A. , Weil, D. , Kalinski, H. , Pel‐Or, Y. , Ayadi, H. , Petit, C. , … Bonne‐Tamir, B. (1997). Mutation profile of all 49 exons of the human myosin VIIA gene, and haplotype analysis in Usher 1B families from diverse origins. American Journal of Human Genetics, 61, 813–821. https://doi.org/10.1086/514899 9382091 10.1086/514899 PMC1716000

[mgg3392-bib-0500] Ahmed, Z. M. , Masmoudi, S. , Kalay, E. , Belyantseva, I. A. , Mosrati, M. A. , Collin, R. W. , … Kremer, H. (2008). Mutations of LRTOMT, a fusion gene with alternative reading frames, cause nonsyndromic deafness in humans. Nat Genet 40:1335‐1340. doi:10.1038/ng.245. 18953341 10.1038/ng.245 PMC3404732

[mgg3392-bib-0007] Aissi‐Ben Moussa, S. , Moussa, A. , Lovecchio, T. , Kourda, N. , Najjar, T. , Ben Jilani, S. , … Buisine, M. P. (2009). Identification and characterization of a novel MLH1 genomic rearrangement as the cause of HNPCC in a Tunisian family: Evidence for a homologous Alu‐mediated recombination. Familial Cancer, 8(2), 119–126. https://doi.org/10.1007/s10689-008-9215-7 18792805 10.1007/s10689-008-9215-7

[mgg3392-bib-0008] Alemanno, M. S. , Cama, E. , Santarelli, R. , Carella, M. , Zelante, L. , Toffolatti, L. , … Arslan, E. (2009). A novel missense mutation in the Connexin 26 gene associated with autosomal recessive nonsyndromic sensorineural hearing loss in a consanguineous Tunisian family. International Journal of Pediatric Otorhinolaryngology, 73(1), 127–131. https://doi.org/10.1016/j.ijporl.2008.09.019 18990456 10.1016/j.ijporl.2008.09.019

[mgg3392-bib-0009] Amouri, A. , Hammami, W. , Kilani, O. , Bouzouita, A. , Ayed, W. , Ben Meftah, M. , … Jaafoura, M. H. (2014). Chromosomal evaluation in a group of Tunisian patients with non‐obstructive azoospermia and severe oligozoospermia attending a Tunisian cytogenetic department. Comptes Rendus Biologies, 337(4), 223–228. https://doi.org/10.1016/j.crvi.2014.02.006 24702890 10.1016/j.crvi.2014.02.006

[mgg3392-bib-0010] Anwar, W. A. , Khyatti, M. , & Hemminki, K. (2014). Consanguinity and genetic diseases in North Africa and immigrants to Europe. European Journal of Public Health, 24(Suppl. 1), 57–63. https://doi.org/10.1093/eurpub/cku104 10.1093/eurpub/cku104 25107999

[mgg3392-bib-0011] Aouadi‐Abdeljaouad, N. , & Belhouchet, L. (2008). Recent prehistoric field research in central Tunisia: Prehistoric occupations in the Meknassy basin. African Archaeological Review, 25, 75–85. https://doi.org/10.1007/s10437-008-9027-z

[mgg3392-bib-0012] Aouadi‐Abdeljaouad, N. , & Belhouchet, L . (2012). Chap 10: Middle Stone Age in Tunisia: Present status of knowledge and recent advances Modern Origins: A North African Perspective (pp. 143–156). https://doi.org/10.1007/978-94-007-2929-2

[mgg3392-bib-0013] Arredi, B. , Poloni, E. S. , Parachini, S. , Zerjal, T. , Fathallah, D. M. , Makfrelouf, M. , … Tyler‐Smith, C. (2004). A predominantly Neolithic origin for Y‐chromosome variation in North Africa. American Journal of Human Genetics, 75, 338–345. https://doi.org/10.1086/423147 15202071 10.1086/423147 PMC1216069

[mgg3392-bib-0014] Azzedine, H. , Bolino, A. , Taïeb, T. , Birouk, N. , Di Duca, M. , Bouhouche, A. , … LeGuern, E. (2003). Mutations in MTMR13, a new pseudophosphatase homologue of MTMR2 and Sbf1, in two families with an autosomal recessive demyelinating form of Charcot‐Marie‐Tooth disease associated with early‐onset glaucoma. American Journal of Human Genetics, 72(5), 1141–1153. https://doi.org/10.1086/375034 12687498 10.1086/375034 PMC1180267

[mgg3392-bib-0015] Barboura, L. , Chebel, S. , Ferchichi, S. , Ben Mansour, R. , Frih‐Ayed, M. , & Miled, A. (2011). Diagnostic strategy of metachromatic leukodystrophy in Tunisia. Annales de Biologie Clinique (Paris), 69(4), 465–469. 10.1684/abc.2011.0599 21896413

[mgg3392-bib-0016] Barhoumi, C. , Amouri, R. , Ben Hamida, C. , Ben Hamida, M. , Machghoul, S. , Gueddiche, M. , & Hentati, F. (2001). Linkage of a new locus for autosomal recessive axonal form of Charcot‐Marie‐Tooth disease to chromosome 8q21.3. Neuromuscular Disorders, 11(1), 27–34. https://doi.org/10.1016/S0960-8966(00)00162-0 11166163 10.1016/s0960-8966(00)00162-0

[mgg3392-bib-0017] Baxter, R. V. , Ben Othmane, K. , Rochelle, J. M. , Stajich, J. E. , Hulette, C. , Dew‐Knight, S. , … Vance, J. M. (2002). Ganglioside‐induced differentiation‐associated protein‐1 is mutant in Charcot‐Marie‐Tooth disease type 4A/8q21. Nature Genetics, 30(1), 21–22. https://doi.org/10.1038/ng796 11743579 10.1038/ng796

[mgg3392-bib-0018] Belguith, H. , Aifa‐Hmani, M. , Dhouib, H. , Said, M. B. , Mosrati, M. A. , Lahmar, I. , … Masmoudi, S. (2009). Screening of the DFNB3 locus: Identification of three novel mutations of MYO15A associated with hearing loss and further suggestion for two distinctive genes on this locus. Genetic Testing and Molecular Biomarkers, 13, 147–151. https://doi.org/10.1089/gtmb.2008.0077 19309289 10.1089/gtmb.2008.0077

[mgg3392-bib-0019] Ben Arab, S. , Masmoudi, S. , Beltaief, N. , Hachicha, S. , & Ayadi, H. (2004). Consanguinity and endogamy in Northern Tunisia and its impact on nonsyndromic deafness. Genetic Epidemiology, 27, 74–79. https://doi.org/10.1002/(ISSN)1098-2272 15185405 10.1002/gepi.10321

[mgg3392-bib-0020] Ben Halim, N. , Ben Alaya Bouafif, N. , Romdhane, L. , Kefi Ben Atig, R. , Chouchane, I. , Bouyacoub, Y. , … Abdelhak, S . (2013). Consanguinity, endogamy, and genetic disorders in Tunisia. Journal of Community Genetics, 4, 273–284. http://www.latunisiemedicale.com/article-medicale-tunisie.php?article=1208 23208456 10.1007/s12687-012-0128-7 PMC3666836

[mgg3392-bib-0021] Ben Halim, N. , Hsouna, S. , Lasram, K. , Rejeb, I. , Walha, A. , Talmoudi, F. , … Abdelhak, S . (2016). Differential impact of consanguineous marriages on autosomal recessive diseases in Tunisia. American Journal of Human Biology, 28, 171–180. https://doi.org/10.1002/ajhb.22764 26179682 10.1002/ajhb.22764

[mgg3392-bib-0022] Ben Hamida, C. , Cavalier, L. , Belal, S. , Sanhaji, H. , Nadal, N. , Barhoumi, C. , … Hentati, F. (1997). Homozygosity mapping of giant axonal neuropathy gene to chromosome 16q24.1. Neurogenetics, 1(2), 129–133. https://doi.org/10.1007/s100480050019 10732815 10.1007/s100480050019

[mgg3392-bib-0023] Ben Hamida, C. , Doerflinger, N. , Belal, S. , Linder, C. , Reutenauer, L. , Dib, C. , … Koenig, M . (1993). Localization of Friedreich ataxia phenotype with selective vitamin E deficiency to chromosome 8q by homozygosity mapping. Nature Genetics, 5(2), 195–200. https://doi.org/10.1038/ng1093-195 8252047 10.1038/ng1093-195

[mgg3392-bib-0024] Ben Hamida, M. , Hentati, F. , & Ben Hamida, C. (1990). Giant axonal neuropathy with inherited multisystem degeneration in a Tunisian kindred. Neurology, 40(2), 245–250. https://doi.org/10.1212/WNL.40.2.245 2153943 10.1212/wnl.40.2.245

[mgg3392-bib-0025] Ben Hamida, M. , & Marrakchi, D . (1980). [Duchenne muscular dystrophy in Tunesia: 31 cases in 13 families with autosomal recessive inheritance]. Journal de Génétique Humaine, 28(1), 1–9. Article in French. 7400780

[mgg3392-bib-0026] Ben Jemaa, L. , Khemir, S. , Maazoul, F. , Richard, L. , Beldjord, C , Chaabouni, M. , & Chaabouni, H . (2008) [Molecular diagnosis of fragile X syndrome]. Tunis Med, 86(11), 973–977. Article in French. 19213487

[mgg3392-bib-0027] Ben Othmane, K. , Ben Hamida, M. , Pericak‐Vance, M. A. , Ben Hamida, C. , Blel, S. , Carter, S. C. , … Vance, J. M. (1992). Linkage of Tunisian autosomal recessive Duchenne‐like muscular dystrophy to the pericentromeric region of chromosome 13q. Nature Genetics, 2(4), 315–317. https://doi.org/10.1038/ng1292-315 1303286 10.1038/ng1292-315

[mgg3392-bib-0028] Ben Othmane, K. , Hentati, F. , Lennon, F. , Ben Hamida, C. , Blel, S. , Roses, A. D. , … Vance, J. M. (1993). Linkage of a locus (CMT4A) for autosomal recessive Charcot‐Marie‐Tooth disease to chromosome 8q. Human Molecular Genetics, 2(10), 1625–1628. https://doi.org/10.1093/hmg/2.10.1625 8268915 10.1093/hmg/2.10.1625

[mgg3392-bib-0029] Ben Othmane, K. , Johnson, E. , Menold, M. , Graham, F. L. , Hamida, M. B. , Hasegawa, O. , … Vance, J. M. (1999). Identification of a new locus for autosomal recessive Charcot‐Marie‐Tooth disease with focally folded myelin on chromosome 11p15. Genomics, 62, 344–349. https://doi.org/10.1006/geno.1999.6028 10644431 10.1006/geno.1999.6028

[mgg3392-bib-0030] Ben Rebeh, I. , Benzina, Z. , Dhouib, H. , Hadjamor, I. , Amyere, M. , Ayadi, L. , … Masmoudi, S. (2008). Identification of candidate regions for a novel Usher syndrome type II locus. Molecular Vision, 14, 1719–1726. 18806881 PMC2538493

[mgg3392-bib-0031] Ben Rebeh, I. , Morinière, M. , Ayadi, L. , Benzina, Z. , Charfedine, I. , Feki, J. , … Masmoudi, S. (2008). Reinforcement of a minor alternative splicing event in MYO7A due to a missense mutation results in a mild form of retinopathy, deafness and vestibular areflexia. Molecular Vision, 14, 1719–1726. 21031134 PMC2956701

[mgg3392-bib-0032] Ben Rekaya, M. , Jerbi, M. , Messaoud, O. , Ben Brick, A. S. , Zghal, M. , Mbarek, C. , … Abdelhak, S. (2013). Further evidence of mutational heterogeneity of XPC gene in Tunisian families: a spectrum of private and ethnic specific mutations. BioMed Research International, 2013, 316286 https://doi.org/10.1155/2013/316286 23984341 10.1155/2013/316286 PMC3741899

[mgg3392-bib-0033] Ben Rekaya, M. , Laroussi, N. , Messaoud, O. , Jones, M. , Jerbi, M. , Naouali, C. , … Yacoub‐Youssef, H. (2014). A founder large deletion mutation in Xeroderma pigmentosum‐Variant form in Tunisia: Implication for molecular diagnosis and therapy. BioMed Research International, 2014, 256245 https://doi.org/10.1155/2014/256245 24877075 10.1155/2014/256245 PMC4024419

[mgg3392-bib-0034] Ben Rekaya, M. , Messaoud, O. , Mebazaa, A. , Riahi, O. , Azaiez, H. , Kefi, R. , … Mokni, M. (2011). A novel POLH gene mutation in a Xeroderma pigmentosum‐V Tunisian patient: Phenotype‐genotype correlation. Journal of Genetics, 90, 483–487. https://doi.org/10.1007/s12041-011-0101-y 22227937 10.1007/s12041-011-0101-y

[mgg3392-bib-0035] Ben Rekaya, M. , Messaoud, O. , Talmoudi, F. , Nouira, S. , Ouragini, H. , Amouri, A. , … Zghal, M. (2009). High frequency of the V548AfsX572 XPC mutation in Tunisia: Implication for molecular diagnosis. Journal of Human Genetics, 54, 426–429. https://doi.org/10.1038/jhg.2009.50 19478817 10.1038/jhg.2009.50

[mgg3392-bib-0036] Ben Rekaya, M. , Naouali, C. , Messaoud, O. , Jones, M. , Bouyacoub, Y. , Nagara, M. , … Abdelhak, S. (2017). Whole Exome Sequencing allows the identification of two novel groups of Xeroderma pigmentosum in Tunisia, XP‐D and XP‐E: Impact on molecular diagnosis. Journal of Dermatological Science, 89(2), 172–180. https://doi.org/10.1016/j.jdermsci.2017.10.015 29169765 10.1016/j.jdermsci.2017.10.015

[mgg3392-bib-0037] Ben Rhouma, B. , Kallabi, F. , Mahfoudh, N. , Ben Mahmoud, A. , Engeli, R. T. , Kamoun, H. , … Belguith, N . (2017). Novel cases of Tunisian patients with mutations in the gene encoding 17β‐hydroxysteroid dehydrogenase type 3 and a founder effect. Journal of Steroid Biochemistry and Molecular Biology, 165(Pt A), 86–94. https://doi.org/10.1016/j.jsbmb.2016.03.007 26956191 10.1016/j.jsbmb.2016.03.007

[mgg3392-bib-0038] Ben Rhouma, F. , Kallel, F. , Kefi, R. , Cherif, W. , Nagara, M. , Azaiez, H. , … Mseddi, S. (2012). Adult Gaucher disease in southern Tunisia: Report of three cases. Diagnostic Pathology, 7, 4 https://doi.org/10.1186/1746-1596-7-4 22233685 10.1186/1746-1596-7-4 PMC3275535

[mgg3392-bib-0039] Ben Said, M. , Grati, M. , Ishimoto, T. , Zou, B. , Chakchouk, I. , Ma, Q. , … Liu, X. (2016). A mutation in SLC22A4 encoding an organic cation transporter expressed in the cochlea strial endothelium causes human recessive non‐syndromic hearing loss DFNB60. Human Genetics, 135(5), 513–524. https://doi.org/10.1007/s00439-016-1657-7 27023905 10.1007/s00439-016-1657-7 PMC4836961

[mgg3392-bib-0040] Ben Said, M. , Hmani‐Aifa, M. , Amar, I. , Baig, S. M. , Mustapha, M. , Delmaghani, S. , … Masmoudi, S. (2010). High frequency of the p. R34X mutation in the TMC1 gene associated with nonsyndromic hearing loss is due to founder effects. Genetic Testing and Molecular Biomarkers, 14, 307–311. https://doi.org/10.1089/gtmb.2009.0174 20373850 10.1089/gtmb.2009.0174 PMC2936956

[mgg3392-bib-0041] Ben Salah, G. , Salem, I. H. , Masmoudi, A. , Ben Rhouma, B. , Turki, H. , Fakhfakh, F. , … Kamoun, H. (2014). Chromosomal instability associated with a novel BLM frameshift mutation (c.1980‐1982delAA) in two unrelated Tunisian families with Bloom syndrome. Journal of the European Academy of Dermatology and Venereology, 10, 1318–1323. https://doi.org/10.1111/jdv.12279 10.1111/jdv.12279 24118499

[mgg3392-bib-0042] Ben Salah, G. , Salem, I. H. , Masmoudi, A. , Kallabi, F. , Turki, H. , Fakhfakh, F. , … Kamoun, H. (2014). A novel frameshift mutation in BLM gene associated with high sister chromatid exchanges (SCE) in heterozygous family members. Molecular Biology Reports, 41, 7373–7380. https://doi.org/10.1007/s11033-014-3624-5 25129257 10.1007/s11033-014-3624-5

[mgg3392-bib-0043] Ben Turkia, H. , Tebib, N. , Azzouz, H. , Abdelmoula, M. S. , Ben Chehida, A. , Chemli, J. , … Ben Dridi, M. F. (2009). Incidence of mucopolysaccharidoses in Tunisia. Tunis Med, 87, 782–785. 20209839

[mgg3392-bib-0044] Benhaj Mbarek, I. , Abroug, S. , Omezzine, A. , Zellama, D. , Achour, A. , Harbi, A. , & Bouslama, A. (2011). Selected AGXT gene mutations analysis provides a genetic diagnosis in 28% of Tunisian patients with primary hyperoxaluria. BMC Nephrology, 12, 25 https://doi.org/10.1186/1471-2369-12-25 21612638 10.1186/1471-2369-12-25 PMC3123632

[mgg3392-bib-0045] Ben‐Mustapha, I. , Ben‐Ali, M. , Mekki, N. , Patin, E. , Harmant, C. , Bouguila, J. , … Barbouche, M. R. (2014). A 1,100‐year‐old founder effect mutation in IL12B gene is responsible for Mendelian susceptibility to mycobacterial disease in Tunisian patients. Immunogenetics, 66(1), 67–71. https://doi.org/10.1007/s00251-013-0739-0 24127073 10.1007/s00251-013-0739-0

[mgg3392-bib-0046] Ben‐Rebeh, I. , Grati, M. , Bonnet, C. , Bouassida, W. , Hadjamor, I. , Ayadi, H. , … Masmoudi, S. (2016). Genetic analysis of Tunisian families with Usher syndrome type 1: Toward improving early molecular diagnosis. Molecular Vision, 22, 827–835. 27440999 PMC4950652

[mgg3392-bib-0047] Bercovich, D. , Elimelech, A. , Zlotogora, J. , Korem, S. , Yardeni, T. , Gal, N. , … Anikster, Y. (2008). Genotype‐phenotype correlations analysis of mutations in the phenylalanine hydroxylase (PAH) gene. Journal of Human Genetics, 53(5), 407–418. https://doi.org/10.1007/s10038-008-0264-4 18299955 10.1007/s10038-008-0264-4

[mgg3392-bib-0048] Bomont, P. , Cavalier, L. , Blondeau, F. , Ben Hamida, C. , Belal, S. , Tazir, M. , … Koenig, M. (2000). The gene encoding gigaxonin, a new member of the cytoskeletal BTB/kelch repeat family, is mutated in giant axonal neuropathy. Nature Genetics, 26(3), 370–374. https://doi.org/10.1038/81701 11062483 10.1038/81701

[mgg3392-bib-0049] Bönnemann, C. G. , Wong, J. , Ben Hamida, C. , Hamida, M. B. , Hentati, F. , & Kunkel, L. M. (1998). LGMD 2E in Tunisia is caused by a homozygous missense mutation in beta‐sarcoglycan exon 3. Neuromuscular Disorders, 8(3–4), 193–197. https://doi.org/10.1016/S0960-8966(98)00014-5 9631401 10.1016/s0960-8966(98)00014-5

[mgg3392-bib-0050] Bouchlaka, C. , Abdelhak, S. , Amouri, A. , Ben Abid, H. , Hadiji, S. , Frikha, M. , … Tunisian Fanconi Anemia Study Group . (2003). Fanconi anemia in Tunisia: High prevalence of group A and identification of new FANCA mutations. Journal of Human Genetics, 48, 352–361. https://doi.org/10.1007/s10038-003-0037-z 12827451 10.1007/s10038-003-0037-z

[mgg3392-bib-0051] Boudaya, M. , Fredj, S. H. , Siala, H. , Bibi, A. , & Messaoud, T. (2013). Identification of a cystic fibrosis mutation W19X in Tunisia. Annales de Biologie Clinique (Paris), 2, 223–226. 10.1684/abc.2013.0790 23587593

[mgg3392-bib-0052] Bouhjar, I. B. , Gmidène, A. , Soyah, N. , Hanene, H. , Mougou, S. , Elghezal, H. , & Saad, A. (2012). Trisomy and tetrasomy 15q11‐q13 diagnosed by molecular cytogenetic analysis in two patients with mental retardation. Journal of Pediatric Genetics, 1(1), 63–68. 27625804 10.3233/PGE-2012-012 PMC5020922

[mgg3392-bib-0053] Boukhris, A. , Feki, I. , Denis, E. , Miladi, M. I. , Brice, A. , Mhiri, C. , & Stevanin, G. (2008). Spastic paraplegia 15: Linkage and clinical description of three Tunisian families. Movement Disorders, 23(3), 429–433. https://doi.org/10.1002/(ISSN)1531-8257 18098276 10.1002/mds.21848

[mgg3392-bib-0054] Boukhris, A. , Feki, I. , Elleuch, N. , Miladi, M. I. , Boland‐Augé, A. , Truchetto, J. , … Stevanin, G . (2010). A new locus (SPG46) maps to 9p21.2‐q21.12 in a Tunisian family with a complicated autosomal recessive hereditary spastic paraplegia with mental impairment and thin corpus callosum. Neurogenetics, 11(4), 441–448. https://doi.org/10.1007/s10048-010-0249-2 20593214 10.1007/s10048-010-0249-2

[mgg3392-bib-0055] Boukhris, A. , Stevanin, G. , Feki, I. , Denora, P. , Elleuch, N. , Miladi, M. I. , … Mhiri, C. (2009). Tunisian hereditary spastic paraplegias: Clinical variability supported by genetic heterogeneity. Clinical Genetics, 75(6), 527–536. https://doi.org/10.1111/j.1399-0004.2009.01176.x 19438933 10.1111/j.1399-0004.2009.01176.x

[mgg3392-bib-0056] Bouyacoub, Y. , Zribi, H. , Azzouz, H. , Nasrallah, F. , Abdelaziz, R. B. , Kacem, M. , … Abdelhak, S. (2013). Novel and recurrent mutations in the TAT gene in Tunisian families affected with Richner‐Hanhart syndrome. Gene, 529(1), 45–49. https://doi.org/10.1016/j.gene.2013.07.066 23954227 10.1016/j.gene.2013.07.066

[mgg3392-bib-0057] Brett, M. , & Fentress, E. (1997). The Berbers. Oxford: Blackwell publishers.

[mgg3392-bib-0058] Campbell, C. L. , Palamara, P. F. , Dubrovsky, M. , Botigué, L. R. , Fellous, M. , Atzmon, G. , … Ostrer, H. (2012). North African Jewish and non‐Jewish populations form distinctive, orthogonal clusters. Proceedings of the National Academy of Sciences of the United States of America, 109(34), 13865–13870. https://doi.org/10.1073/pnas.1204840109 22869716 10.1073/pnas.1204840109 PMC3427049

[mgg3392-bib-0059] Cavalier, L. , Ben Hamida, C. , Amouri, R. , Belal, S. , Bomont, P. , Lagarde, N. , … Hentati, F. (2000). Giant axonal neuropathy locus refinement to a < 590 kb critical interval. European Journal of Human Genetics, 8(7), 527–534. https://doi.org/10.1038/sj.ejhg.5200476 10909853 10.1038/sj.ejhg.5200476

[mgg3392-bib-0060] Chaabouni, H. , Chaabouni, M. , Maazoul, F. , M'Rad, R. , Jemaa, L. B. , Smaoui, N. , … Zouari, F. (2001). Prenatal diagnosis of chromosome disorders in Tunisian population. Annales de Genetique, 44(2), 99–104. https://doi.org/10.1016/S0003-3995(01)01046-2 11522249 10.1016/s0003-3995(01)01046-2

[mgg3392-bib-0061] Chaabouni, H. , Nemsia, J. , Riou, S. , Largueche, S. , & Ferchiou, A . (1986). Les malformations congénitales: Dépistage néonatal dans une maternité tunisienne. Maghreb Med, 129, 49–54. Article in French.

[mgg3392-bib-0062] Chaabouni‐Bouhamed, H. (2008). Tunisia: Communities and community genetics. Community Genetics, 11(6), 313–323. https://doi.org/10.1159/000133303 18689999 10.1159/000133303

[mgg3392-bib-0063] Chahed, M. K. , & Arfa, C . (2014). Monitoring and evaluating progress towards Universal Health Coverage in Tunisia. PLoS Medicine, 11(9), e1001729. https://doi.org/10.1371/journal.pmed.1001729 eCollection 2014 Sep. 10.1371/journal.pmed.1001729 PMC4170955 25243673

[mgg3392-bib-0064] Chakchouk, I. , Ben Said, M. , Jbeli, F. , Benmarzoug, R. , Loukil, S. , Smeti, I. , … Masmoudi, S. (2015). NADf Chip, a two‐color microarray for simultaneous screening of multigene mutations associated with hearing impairment in North African Mediterranean Countries. Journal of molecular diagnostics, 17(2), 155–161. https://doi.org/10.1016/j.jmoldx.2014.11.003 25560255 10.1016/j.jmoldx.2014.11.003

[mgg3392-bib-0065] Chakchouk, I. , Grati, M. , Bademci, G. , Bensaid, M. , Ma, Q. , Chakroun, A. , … Liu, X.Z . (2015). Novel mutations confirm that COL11A2 is responsible for autosomal recessive non‐syndromic hearing loss DFNB53. Molecular Genetics and Genomics, 290(4), 1327–1334. https://doi.org/10.1007/s00438-015-0995-9 25633957 10.1007/s00438-015-0995-9 PMC4707654

[mgg3392-bib-0066] Charfeddine, I. , Mnejja, M. , Hammami, B. , Chakroun, A. , Masmoudi, S. , Ayadi, H. , & Ghorbel, A. (2010). Pendred syndrome in Tunisia. European Annals of Otorhinolaryngology, HEAD and Neck Diseases, 127(1), 7–10. https://doi.org/10.1016/j.anorl.2010.02.002 10.1016/j.anorl.2010.02.002 20822748

[mgg3392-bib-0067] Charfeddine, C. , Monastiri, K. , Mokni, M. , Laadjimi, A. , Kaabachi, N. , Perin, O. , … Abdelhak, S. (2006). Clinical and mutational investigations of tyrosinemia type II in Northern Tunisia: Identification and structural characterization of two novel TAT mutations. Molecular Genetics and Metabolism, 88(2), 184–191. https://doi.org/10.1016/j.ymgme.2006.02.006 16574453 10.1016/j.ymgme.2006.02.006

[mgg3392-bib-0068] Charoute, H. , Bakhchane, A. , Benrahma, H. , Romdhane, L. , Gabi, K. , Rouba, H. , … Barakat, A. (2015). Mediterranean founder mutation database (MFMD): Taking advantage from founder mutations in genetics diagnosis, genetic diversity and migration history of the Mediterranean population. Human Mutation, 36(11), E2441–E2453. https://doi.org/10.1002/humu.22835 26173767 10.1002/humu.22835

[mgg3392-bib-0069] Chelli, D. , Dimassi, K. , Chaabouni, M. , Ben Saad, M. , Mssaed, H. , Bchir, F. , … Chanoufi, B . (2008). [Prenatal diagnosis of trisomy 21: The Tunisian experience]. Sante, 18(4), 199–203. Article in French. 19810614

[mgg3392-bib-0070] Cherif, W. , Ben Turkia, H. , Ben Rhouma, F. , Riahi, I. , Chemli, J. , Kefi, R. , … Abdelhak, S. (2009). Gaucher disease in Tunisia: High frequency of the most common mutations. Blood Cells, Molecules, & Diseases, 43, 161–162. https://doi.org/10.1016/j.bcmd.2009.05.004 10.1016/j.bcmd.2009.05.004 19553144

[mgg3392-bib-0071] Chkioua, L. , Khedhiri, S. , Ben Turkia, H. , Chahed, H. , Ferchichi, S. , Ben Dridi, M. F. , … Miled, A. (2011). Hurler disease (mucopolysaccharidosis type IH): Clinical features and consanguinity in Tunisian population. Diagnostic Pathology, 6, 113 https://doi.org/10.1186/1746-1596-6-113 22074387 10.1186/1746-1596-6-113 PMC3261812

[mgg3392-bib-0072] Chkioua, L. , Khedhiri, S. , Ferchichi, S. , Tcheng, R. , Chahed, H. , Froissart, R. , … Miled, A. (2011). Molecular analysis of iduronate‐2‐sulfatase gene in Tunisian patients with MPS type II. Diagnostic Pathology, 6, 42 https://doi.org/10.1186/1746-1596-6-42 21605424 10.1186/1746-1596-6-42 PMC3115838

[mgg3392-bib-0073] Chkioua, L. , Khedhiri, S. , Grissa, O. , Aloui, C. , Ben Turkia, H. , Ferchichi, S. , … Laradi, S. (2015). Genetic basis of cystinosis in Tunisian patients: Identification of novel mutation in CTNS gene. Meta Gene, 5, 144–149. https://doi.org/10.1016/j.mgene.2015.07.003 26266097 10.1016/j.mgene.2015.07.003 PMC4528043

[mgg3392-bib-0074] Chkioua, L. , Khedhiri, S. , Hafsi, H. , Grissa, O. , Ben turkia, H. , Miled, A. , … Alif, N . (2016). Molecular analysis in a GALNS study cohort of 15 Tunisian patients: Description of a novel mutation. Diagnostic Pathology, 11 (1), 51 https://doi.org/10.1186/s13000-016-0498-y 27317439 10.1186/s13000-016-0498-y PMC4912732

[mgg3392-bib-0075] Chkioua, L. , Khedhiri, S. , Jaidane, Z. , Ferchichi, S. , Habib, S. , Froissart, R. , … Laradi, S. (2007). Mucopolysaccharidosis type I: Identification of alpha‐L‐iduronidase mutations in Tunisian families. Archives de Pediatrie, 14, 1183–1189. https://doi.org/10.1016/j.arcped.2007.06.018 17728118 10.1016/j.arcped.2007.06.018

[mgg3392-bib-0076] Chkioua, L. , Khedhiri, S. , Kassab, A. , Bibi, A. , Ferchichi, S. , Froissart, R. , … Miled, A. (2011). Molecular analysis of mucopolysaccharidosis type I in Tunisia: Identification of novel mutation and eight Novel polymorphisms. Diagnostic Pathology, 6, 39 https://doi.org/10.1186/1746-1596-6-39 21521498 10.1186/1746-1596-6-39 PMC3110106

[mgg3392-bib-0077] Chouchane, L. , & Hassen, E . (2010). Chap 21: Genetic disorders in Tunisia Genetic disorders among Arab populations (pp. 613–638). Second Edition, Teebi, A.S. https://doi.org/10.1007/978-3-642-05080-0

[mgg3392-bib-0078] Chouk, I. , Daoud, B. B. , Mellouli, F. , Bejaoui, M. , G′erard, N. , & Dellagi, K. (2004). Contribution to the description of the beta‐thalassemia spectrum in Tunisia and the origin of mutation diversity. Hemoglobin, 28, 189–195. https://doi.org/10.1081/HEM-120040305 15481885 10.1081/hem-120040305

[mgg3392-bib-0079] Dandana, A. , Ben Khelifa, S. , Chahed, H. , Miled, A. , & Ferchichi, S. (2016). Gaucher disease: Clinical, biological and therapeutic aspects. Pathobiology, 83(1), 13–23. https://doi.org/10.1159/000440865 26588331 10.1159/000440865

[mgg3392-bib-0080] Daoud, B. B. , Mosbehim, I. , Prehu, C. , Chaouachi, D. , Hafsia, R. , & Abbes, S. (2008). Molecular characterization of erythrocyte glucose‐6‐phosphate dehydrogenase deficiency in Tunisia. Pathologie Biologie, 56(5), 260–267. https://doi.org/10.1016/j.patbio.2007.08.009 18226470 10.1016/j.patbio.2007.08.009

[mgg3392-bib-0081] Driss, A. , Amouri, R. , Ben Hamida, C. , Souilem, S. , Gouider‐Khouja, N. , Ben Hamida, M. , & Hentati, F. (2000). A new locus for autosomal recessive limb‐girdle muscular dystrophy in a large consanguineous Tunisian family maps to chromosome 19q13.3. Neuromuscular Disorders, 10(4–5), 240–246. https://doi.org/10.1016/S0960-8966(00)00099-7 10838249 10.1016/s0960-8966(00)00099-7

[mgg3392-bib-0082] El Euch‐Fayache, G. , Bouhlal, Y. , Amouri, R. , Feki, M. , & Hentati, F. (2014). Molecular, clinical and peripheral neuropathy study of Tunisian patients with ataxia with vitamin E deficiency. Brain, 137(Pt 2), 402–410. https://doi.org/10.1093/brain/awt339 24369383 10.1093/brain/awt339

[mgg3392-bib-0083] El Moncer, W. , Esteban, E. , Bahri, R. , Gayà‐Vidal, M. , Carreras‐Torres, R. , Athanasiadis, G. , … Chaabani, H. (2010). Mixed origin of the current Tunisian population from the analysis of Alu and Alu/STR compound systems. Journal of Human Genetics, 55(12), 827–833. https://doi.org/10.1038/jhg.2010.120 20882034 10.1038/jhg.2010.120

[mgg3392-bib-0084] Elleuch, M. , Mnif Feki, M. , Kammoun, M. , Charfi, N. , Rekik, N. , Bouraoui, A. , … Abid, M. (2010). Descriptive analyses of Turner syndrome: 49 cases in Tunisia. Annales Endocrinol (Paris), 71(2), 111–116. https://doi.org/10.1016/j.ando.2009.12.013 10.1016/j.ando.2009.12.013 20153455

[mgg3392-bib-0085] Elloumi‐Zghal, H. , Barbouche, M. R. , Chemli, J. , Béjaoui, M. , Harbi, A. , Snoussi, N. , … Dellagi, K. (2002). Clinical and genetic heterogeneity of inherited autosomal recessive susceptibility to disseminated *Mycobacterium bovis* bacille calmette‐guérin infection. Journal of Infectious Diseases, 185(10), 1468–1475. https://doi.org/10.1086/340510 11992283 10.1086/340510

[mgg3392-bib-0086] Fadhlaoui‐Zid, K. , Martinez‐Cruz, B. , Khodjet‐el‐khil, H. , Mendizabal, I. , Benammar‐Elgaaied, A. , & Comas, D. (2011). Genetic structure of Tunisian ethnic groups revealed by paternal lineages. American Journal of Physical Anthropology, 146, 271–280. https://doi.org/10.1002/ajpa.21581 21915847 10.1002/ajpa.21581

[mgg3392-bib-0087] Fadhlaoui‐Zid, K. , Plaza, S. , Calafell, F. , Ben Amor, M. , Comas, D. , & Bennamar El Gaaied, A. (2004). Mitochondrial DNA heterogeneity in Tunisian Berbers. Annals of Human Genetics, 68, 222–233. https://doi.org/10.1046/j.1529-8817.2004.00096.x 15180702 10.1046/j.1529-8817.2004.00096.x

[mgg3392-bib-0088] Falik‐Zaccai, T. C. , Shachak, E. , Yalon, M. , Lis, Z. , Borochowitz, Z. , Macpherson, J. N. , … Eichler, E. E. (1997). Predisposition to the fragile X syndrome in Jews of Tunisian descent is due to the absence of AGG interruptions on a rare Mediterranean haplotype. American Journal of Human Genetics, 60(1), 103–112. 8981953 PMC1712540

[mgg3392-bib-0089] Fattoum, S. (2006). Hemoglobinopathies in Tunisia. An updated review of the epidemiologic and molecular data. Tunis Med, 84, 687–696. 17294892

[mgg3392-bib-0090] Ferembach, D. (1985). On the origin of the Iberomaurusians. A new hypothesis. Journal of Human Evolution, 14, 393–397. https://doi.org/10.1016/S0047-2484(85)80047-6

[mgg3392-bib-0091] Fernandez Vina, M. A. , Hollenbach, J. A. , Lyke, K. E. , Sztein, M. B. , Maiers, M. , Klitz, W. , … Cao, K. (2012). Tracking human migrations by the analysis of the distribution of HLA alleles, lineages and haplotypes in closed and open populations. Philosophical Transactions of the Royal Society of London. Series B, Biological sciences, 367, 820–829. https://doi.org/10.1098/rstb.2011.0320 22312049 10.1098/rstb.2011.0320 PMC3267126

[mgg3392-bib-0092] Fredj, S. H. , Messaoud, T. , Templin, C. , des Georges, M. , Fattoum, S. , & Claustres, M. (2009). Cystic fibrosis transmembrane conductance regulator mutation spectrum in patients with cystic fibrosis in Tunisia. Genetic Testing and Molecular Biomarkers, 13, 577–581. https://doi.org/10.1089/gtmb.2009.0028 19715466 10.1089/gtmb.2009.0028

[mgg3392-bib-0093] Frigi, S. , Cherni, L. , Fadhlaoui‐Zid, K. , & Benammar‐Elgaaied, A. (2010). Ancient local evolution of African mtDNA haplogroups in Tunisian Berber populations. Human Biology, 82, 367–384. https://doi.org/10.3378/027.082.0402 21082907 10.3378/027.082.0402

[mgg3392-bib-0094] Ghorbel, M. , Gargouri Baklouti, S. , Ben Abdallah, F. , Zribi, N. , Cherif, M. , Keskes, R. , … Ammar‐Keskes, L. (2012). Chromosomal defects in infertile men with poor semen quality. Journal of Assisted Reproduction and Genetics, 29(5), 451–456. https://doi.org/10.1007/s10815-012-9737-7 22406877 10.1007/s10815-012-9737-7 PMC3348275

[mgg3392-bib-0095] Gilad, S. , Bar‐Shira, A. , Harnik, R. , Shkedy, D. , Ziv, Y. , Khosravi, R. , … Shiloh, Y. (1996). Ataxia‐telangiectasia: Founder effect among North African Jews. Human Molecular Genetics, 5, 2033–2037. https://doi.org/10.1093/hmg/5.12.2033 8968760 10.1093/hmg/5.12.2033

[mgg3392-bib-0096] Gragueb, A. (1980). Un nouveau gisement acheuleen dans la vallee de l'Oued Mellegue (Nord –ouest de la Tunisie. L'Anthropology (Paris), 84, 359–379.

[mgg3392-bib-0097] Grati, M. , Chakchouk, I. , Ma, Q. , Bensaid, M. , Desmidt, A. , Turki, N. , … Driss, N. (2015). A missense mutation in *DCDC2* causes human recessive deafness DFNB66, likely by interfering with sensory hair cell and supporting cell cilia length regulation. Human Molecular Genetics, 24, 2482–2491. https://doi.org/10.1093/hmg/ddv009 25601850 10.1093/hmg/ddv009 PMC4383862

[mgg3392-bib-0098] Gritli, S. , Omar, S. , Tartaglini, E. , Guannouni, S. , Fleming, J. C. , Steinkamp, M. P. , … Neufeld, E. J. (2001). A novel mutation in the SLC19A2 gene in a Tunisian family with thiamine‐responsive megaloblastic anaemia, diabetes and deafness syndrome. British Journal of Haematology, 113(2), 508–513. https://doi.org/10.1046/j.1365-2141.2001.02774.x 11380424 10.1046/j.1365-2141.2001.02774.x

[mgg3392-bib-0099] Gruet, M . (1954). Le gisement mousterien d'El‐Guettar. Karthago (Paris), 5:1–79. (Article in French)

[mgg3392-bib-0100] Guellouz, N. , Ben Mansour, I. , Ouederni, M. , Jabnoun, S. , Kacem, S. , Mokrani, C. , … Khrouf, N. (2010). Neonatal screening of G6PD deficiency in Tunisia. Archives de l'Institut Pasteur de Tunis, 87, 69–76. 21604461

[mgg3392-bib-0101] Guilford, P. , Ben Arab, S. , Blanchard, S. , Levilliers, J. , Weissenbach, J. , Belkahia, A. , & Petit, C. (1994). A non‐syndrome form of neurosensory, recessive deafness maps to the pericentromeric region of chromosome 13q. Nature Genetics, 6(1), 24–28. https://doi.org/10.1038/ng0194-24 8136828 10.1038/ng0194-24

[mgg3392-bib-0102] Hadiji Mseddi, S. , Kammoun, L. , Bellaaj, H. , Ben Youssef, Y. , Aissaoui, L. , & Torjemane, L. , … le Groupe d'etude tunisien de l'anemie de Fanconi GETAF . (2012). [Creation and report of the Tunisian Fanconi Anemia Registry (TFAR)]. Archives de Pediatrie, 19, 467–475. Article in french https://doi.org/10.1016/j.arcped.2012.02.017 22480464 10.1016/j.arcped.2012.02.017

[mgg3392-bib-0103] Hadj Fredj, S. , Boudaya, M. , Oueslati, S. , Sahnoun, S. , Sahli, C. , Siala, H. , … Messaoud, T . (2013). New frameshift CF mutation 3729delAinsTCT in a Tunisian cystic fibrosis patient. Journal of Genetics, 92(1), 81–83. https://doi.org/10.1007/s12041-013-0208-4 23640408 10.1007/s12041-013-0208-4

[mgg3392-bib-0104] Hadj Fredj, S. , Ouali, F. , Siala, H. , Bibi, A. , Othmani, R. , Dakhlaoui, B. , … Messaoud, T. (2015). Prenatal diagnosis of cystic fibrosis: 10‐years experience. Pathologie Biologie, 63(3), 126–129. https://doi.org/10.1016/j.patbio.2015.04.002 26002249 10.1016/j.patbio.2015.04.002

[mgg3392-bib-0105] Hadj‐Taieb, S. , Nasrallah, F. , Hammami, M. B. , Elasmi, M. , Sanhaji, H. , Moncef, F. , & Kaabachi, N. (2012). Aminoacidopathies and organic acidurias in Tunisia: A retrospective survey over 23 years. Tunis Med, 90(3), 258–261. 22481200

[mgg3392-bib-0106] Haj Khelil, A. , Denden, S. , Leban, N. , Daimi, H. , Lakhdhar, R. , Lefranc, G. , … Perrin, P. (2010). Hemoglobinopathies in North Africa: A review. Hemoglobin, 34(1), 1–23. https://doi.org/10.3109/03630260903571286 20113284 10.3109/03630260903571286

[mgg3392-bib-0107] Haj Khelil, A. , Laradi, S. , Miled, A. , Tadmouri, G. O. , Ben Chibani, J. , & Perrin, P. (2004). Clinical and molecular aspects of hemoglobinopathies in Tunisia. Clinica Chimica Acta, 340, 127–137. https://doi.org/10.1016/j.cccn.2003.10.022 10.1016/j.cccn.2003.10.022 14734204

[mgg3392-bib-0108] Hajjej, A. , Almawi, W. Y. , Hattab, L. , El‐Gaaied, A. , & Hmida, S. (2015). HLA class I and class II alleles and haplotypes confirm the Berber Origin of the Present Day Tunisian Population. PLoS One, 10(8), e0136909 https://doi.org/10.1371/journal.pone.0136909 26317228 10.1371/journal.pone.0136909 PMC4552629

[mgg3392-bib-0109] Hajjej, A. , Almawi, W. Y. , Hattab, L. , El‐Gaaied, A. , & Hmida, S. (2016). The investigation of the origin of Southern Tunisians using HLA genes. Journal of Human Genetics, 62(3), 419–429. 27881842 10.1038/jhg.2016.146

[mgg3392-bib-0110] Hajjej, A. , Hajjej, G. , Almawi, W. Y. , Kaabi, H. , El‐Gaaied, A. , & Hmida, S. (2011). HLA class I and class II polymorphism in a population from south‐eastern Tunisia (Gabes Area). International Journal of Immunogenetics, 38, 191–199. https://doi.org/10.1111/j.1744-313X.2011.01003.x 21385325 10.1111/j.1744-313X.2011.01003.x

[mgg3392-bib-0111] Hajjej, A. , Hmida, S. , Kaabi, H. , Dridi, A. , Jridi, A. , El Gaaled, A. , & Boukef, K. (2006). HLA genes in Southern Tunisians (Ghannouch area) and their relationship with other Mediterraneans. European Journal of Medical Genetics, 49, 43–56. https://doi.org/10.1016/j.ejmg.2005.01.001 16473309 10.1016/j.ejmg.2005.01.001

[mgg3392-bib-0112] Hajjej, A. , Sellami, M. H. , Kaabi, H. , Hajjej, G. , El‐Gaaied, A. , Boukef, K. , … Hmida, S. (2011). HLA class I and class II polymorphisms in Tunisian Berbers. Annals of Human Biology, 38, 156–164. https://doi.org/10.3109/03014460.2010.504195 20666704 10.3109/03014460.2010.504195

[mgg3392-bib-0113] Hajri, S. , Raifman, S. , Gerdts, C. , Baum, S. , & Foster, D. G. (2015). (2015) ‘This Is Real Misery’: Experiences of women denied legal abortion in Tunisia. PLoS One, 10(12), e0145338 https://doi.org/10.1371/journal.pone.0145338 26684189 10.1371/journal.pone.0145338 PMC4686168

[mgg3392-bib-0114] Halioui‐Louhaichi, S. , Ben Chehida, A. , Hassouna, R. , Massaoud, T , Ben Dridi, M.F. , Barsaoui, S. , … Maherzi, A . (2015). [Cystic fibrosis: A report of 33 pediatric Tunisian cases]. Tunis Med, 93(8‐9), 569–573. Article in French. 26815526

[mgg3392-bib-0115] Haloui, S. , Laouini, N. , Sahli, C. A. , Daboubi, R. , Becher, M. , Jouini, L. , … Messaoud, T. (2016). Molecular identification of Gd A‐ and Gd B‐ G6PD deficient variants by ARMS‐PCR in a Tunisian population. Annales de Biologie Clinique, 74, 219–226. 27029726 10.1684/abc.2016.1123

[mgg3392-bib-0116] Hammer, M. B. , Eleuch‐Fayache, G. , Schottlaender, L. V. , Nehdi, H. , Gibbs, J. R. , Arepalli, S. K. , … Singleton, A. B. (2013). Mutations in GBA2 cause autosomal‐recessive cerebellar ataxia with spasticity. American Journal of Human Genetics, 92(2), 245–251. https://doi.org/10.1016/j.ajhg.2012.12.012 23332917 10.1016/j.ajhg.2012.12.012 PMC3567281

[mgg3392-bib-0117] Hanein, S. , Martin, E. , Boukhris, A. , Byrne, P. , Goizet, C. , Hamri, A. , … Stevanin, G. (2008). Identification of the SPG15 gene, encoding spastizin, as a frequent cause of complicated autosomal‐recessive spastic paraplegia, including Kjellin syndrome. American Journal of Human Genetics, 82(4), 992–1002. https://doi.org/10.1016/j.ajhg.2008.03.004 18394578 10.1016/j.ajhg.2008.03.004 PMC2427184

[mgg3392-bib-0118] Hentati, A. , Bejaoui, K. , Pericak‐Vance, M. A. , Hentati, F. , Speer, M. C. , Hung, W. Y. , … Siddique, T . (1994). Linkage of recessive familial amyotrophic lateral sclerosis to chromosome 2q33‐q35. Nature Genetics, 7(3), 425–428. https://doi.org/10.1038/ng0794-425 7920663 10.1038/ng0794-425

[mgg3392-bib-0119] Hermassi, H . (2013). Tunisian revolution and regional imbalance. Journal of Management and Business Studies 2(2), 80–84. http://garj.org/garjmbs/index.htm

[mgg3392-bib-0120] Hmani‐Aifa, M. , Benzina, Z. , Zulfiqar, F. , Dhouib, H. , Shahzadi, A. , Ghorbel, A. , … Ayadi, H. (2009). Identification of two new mutations in the GPR98 and the PDE6B genes segregating in a Tunisian family. European Journal of Human Genetics, 17(4), 474–482. https://doi.org/10.1038/ejhg.2008.167 18854872 10.1038/ejhg.2008.167 PMC2986209

[mgg3392-bib-0121] Hulihan, M. M. , Ishihara‐Paul, L. , Kachergus, J. , Warren, L. , Amouri, R. , Elango, R. , … Farrer, M. J. (2008). LRRK2 Gly2019Ser penetrance in Arab‐Berber patients from Tunisia: A case‐control genetic study. Lancet Neurology, 7, 591–594. https://doi.org/10.1016/S1474-4422(08)70116-9 18539535 10.1016/S1474-4422(08)70116-9

[mgg3392-bib-0122] Jaafar, N. , Moleirinho, A. , Kerkeni, E. , Monastiri, K. , Seboui, H. , Amorim, A. , … Quental, S. (2013). Molecular characterization of maple syrup urine disease patients from Tunisia. Gene, 517(1), 116–119. https://doi.org/10.1016/j.gene.2012.12.097 23313820 10.1016/j.gene.2012.12.097

[mgg3392-bib-0123] Jerbi, M. , Ben Rekaya, M. , Naouali, C. , Jones, M. , Messaoud, O. , Tounsi, H. , … Yacoub‐Youssef, H. (2016). Clinical, genealogical and molecular investigation of the xeroderma pigmentosum type C complementation group in Tunisia. British Journal of Dermatology, 174(2), 439–443. https://doi.org/10.1111/bjd.14046 26211814 10.1111/bjd.14046

[mgg3392-bib-0124] Kammoun, I. , Chaabouni, M. , Trabelsi, M. , Ouertani, I. , Kraoua, L. , Chelly, I. , … Chaabouni, H . (2008). Genetic analysis of Turner syndrome: 89 cases in Tunisia. Annales Endocrinol (Paris), 69(5), 440–445. https://doi.org/10.1016/j.ando.2008.01.007 French. 10.1016/j.ando.2008.01.007 18541220

[mgg3392-bib-0125] Kanoun, H. , Jarraya, F. , Maalej, B. , Lahiani, A. , Mahfoudh, H. , Makni, F. , … Fakhfakh, F. (2017). Identification of compound heterozygous patients with primary hyperoxaluria type 1: Clinical evaluations and in silico investigations. BMC Nephrology, 18(1), 303 https://doi.org/10.1186/s12882-017-0719-y 28969594 10.1186/s12882-017-0719-y PMC5625645

[mgg3392-bib-0126] Kelmemi, W. , Chelly, I. , Kharrat, M. , & Chaabouni, H. (2015). Consanguinity and homozygosity among Tunisian patients with autosomal recessive disorder. Journal of Biosocial Science, 47(6), 718–726. https://doi.org/10.1017/S002193201400056X 25630711 10.1017/S002193201400056X

[mgg3392-bib-0127] Kerem, E. , & Kerem, B . (1995). The relationship between genotype and phenotype in cystic fibrosis. Current Opinion in Pulmonary Medicine, 1(6), 450–456. Review. https://doi.org/10.1097/00063198-199511000-00004 9363081 10.1097/00063198-199511000-00004

[mgg3392-bib-0128] Kharrat, M. , Tardy, V. , M'Rad, R. , Maazoul, F. , Jemaa, L. B. , Refaï, M. , … Chaabouni, H. (2004). Molecular genetic analysis of Tunisian patients with a classic form of 21‐hydroxylase deficiency: Identification of four novel mutations and high prevalence of Q318X mutation. Journal of Clinical Endocrinology and Metabolism, 89(1), 368–374. https://doi.org/10.1210/jc.2003-031056 14715874 10.1210/jc.2003-031056

[mgg3392-bib-0129] Kharrat, M. , Trabelsi, S. , Chaabouni, M. , Maazoul, F. , Kraoua, L. , Ben Jemaa, L. , … Chaabouni, H . (2010). Only two mutations detected in 15 Tunisian patients with 11β‐hydroxylase deficiency: The p.Q356X and the novel p.G379V. Clinical Genetics, 78(4), 398–401. https://doi.org/10.1111/j.1399-0004.2010.01403.x 20331679 10.1111/j.1399-0004.2010.01403.x

[mgg3392-bib-0130] Khedhiri, S. , Chkioua, L. , Bouzidi, H. , Dandana, A. , Ben Turkia, H. , Miled, A. , & Laradi, S. (2009). Mucopolysaccharidoses type I and IVA: Clinical features and consanguinity in Tunisia. Pathologie Biologie, 57, 392–397. https://doi.org/10.1016/j.patbio.2008.05.005 18584975 10.1016/j.patbio.2008.05.005

[mgg3392-bib-0131] Khedhiri, S. , Chkioua, L. , Bouzidi, H. , Dandana, A. , Ferchichi, S. , Ben Turkia, H. , … Laradi, S. (2012). Mucopolysaccharidosis IVA within Tunisian patients: Confirmation of the two novel GALNS gene mutations. Pathologie Biologie, 60, 190–192. https://doi.org/10.1016/j.patbio.2011.03.001 22078177 10.1016/j.patbio.2011.03.001

[mgg3392-bib-0132] Khedhiri, S. , Chkioua, L. , Ferchichi, S. , Bouzidi, H. , Haj Khelil, A. , Ben Mansour, R. , … Laradi, S . (2007). Étude de la maladie de Morquio A dans une famille tunisienne présentant deux enfants atteints. Annales de Biologie Clinique, 65(1), 59–63. Article in french 17264040

[mgg3392-bib-0133] Khedhiri, S. , Chkioua, L. , Ferchichi, S. , Miled, A. , & Laradi, S . (2011). La maladie de Morquio A: Etude Clinique et moleculaire des patients tunisiens. Annales de Biologie Clinique, 69(4), 425–429. Article in French 21896407 10.1684/abc.2011.0593

[mgg3392-bib-0134] Khemir, S. , Tebib, N. , Nasrallah, F. , Ben Nour, F. , Mizouni, H. , Elasmi, M. , … Kaabachi, N. (2009). Phenylketonuria in Tunisian institutions for the mentally handicapped. Archives of Disease in Childhood, 94(8), 647–648. https://doi.org/10.1136/adc.2008.143081. No abstract available 10.1136/adc.2008.143081 19628886

[mgg3392-bib-0135] Khrouf, N. , Spång, R. , Podgorna, T. , Miled, S. B. , Moussaoui, M. , & Chibani, M. (1986). Malformations in 10,000 consecutive births in Tunis. Acta Paediatrica Scandinavica, 75(4), 534–539. https://doi.org/10.1111/j.1651-2227.1986.tb10245.x 3751547 10.1111/j.1651-2227.1986.tb10245.x

[mgg3392-bib-0136] Laouini, N. , Bibi, A. , Ammar, H. , Kazdaghli, K. , Ouali, F. , Othmani, R. , … Messsaoud, T. (2013). Glucose‐6‐phosphate dehydrogenase deficiency in Tunisia: Molecular data and phenotype‐genotype association. Molecular Biology Reports, 40, 851–856. https://doi.org/10.1007/s11033-012-2124-8 23065279 10.1007/s11033-012-2124-8

[mgg3392-bib-0137] Lapidus, I. M. (2002). A history of Islamic societies. Cambridge University Press, 2002, 302–304.

[mgg3392-bib-0138] Laradi, S. , Monastiri, K. , Ferchichi, S. , Nabli, N. , Aouini Rea, P. , Ben Limam, H. , … Maire, I . (2001). Clinico‐biologic and molecular study of mucopolysaccharidosis in Central and Southern Tunisia. Annales de Biologie Clinique, 59, 100–104. Article in French 11252262

[mgg3392-bib-0139] Laradi, S. , Tukel, T. , Erazo, M. , Shabbeer, J. , Chkioua, L. , Khedhiri, S. , … Desnick, R. J. (2005). Mucopolysaccharidosis I Alpha‐LIduronidase mutations in three Tunisian families. Journal of Inherited Metabolic Disease, 28, 1019–1026. https://doi.org/10.1007/s10545-005-0197-4 16435195 10.1007/s10545-005-0197-4

[mgg3392-bib-0140] Lederer, C. W. , Basak, A. N. , Aydinok, Y. , Christou, S. , El‐Beshlawy, A. , Eleftheriou, A. , … Kleanthous, M. (2009). An electronic infrastructure for research and treatment of the thalassemias and other hemoglobinopathies: The Euro‐mediterranean ITHANET project. Hemoglobin, 33(3), 163–176. https://doi.org/10.1080/03630260903089177 19657830 10.1080/03630260903089177

[mgg3392-bib-0141] Lesage, S. , Dürr, A. , Tazir, M. , Lohmann, E. , Leutenegger, A. L. , Janin, S. , … French Parkinson's Disease Genetics Study Group . (2006). LRRK2 G2019S as a cause of Parkinson's disease in North African Arabs. New England Journal of Medicine, 354(4), 422–423. https://doi.org/10.1056/NEJMc055540 16436781 10.1056/NEJMc055540

[mgg3392-bib-0142] Loueslati, B. Y. , Cherni, L. , Khodjet‐Elkhil, H. , Ennafaa, H. , Pereira, L. , Amorim, A. , … Ben Ammar Elgaaied, A. (2006). Islands inside an island: Reproductive isolates on Jerba island. American Journal of Human Biology, 18(1), 149–153. https://doi.org/10.1002/(ISSN)1520-6300 16378336 10.1002/ajhb.20473

[mgg3392-bib-0143] Lucette, V. , & Abraham, L. U . (1991). Juifs en terre d'islam: les communautés de Djerba 13 (éd. Archives contemporaines, Paris). (Article in French)

[mgg3392-bib-0144] Mahfoudh, W. , Bouaouina, N. , Ahmed, S. B. , Gabbouj, S. , Shan, J. , Mathew, R. , … Chouchane, L. (2012). Hereditary breast cancer in Middle Eastern and North African (MENA) populations: Identification of novel, recurrent and founder BRCA1 mutations in the Tunisian population. Molecular Biology Reports, 39(2), 1037–1046. https://doi.org/10.1007/s11033-011-0829-8 21603858 10.1007/s11033-011-0829-8 PMC3249560

[mgg3392-bib-0145] Makhloufi, K. , Ventelou, B. , & Abu‐Zaineh, M. (2015). Have health insurance reforms in Tunisia attained their intended objectives? International Journal of Health Economics and Management, 15(1), 29–51. https://doi.org/10.1007/s10754-014-9157-6 27878666 10.1007/s10754-014-9157-6

[mgg3392-bib-0146] Martin, E. , Schüle, R. , Smets, K. , Rastetter, A. , Boukhris, A. , Loureiro, J. L. , … Stevanin, G. (2013). Loss of function of glucocerebrosidase GBA2 is responsible for motor neuron defects in hereditary spastic paraplegia. American Journal of Human Genetics, 92(2), 238–244. https://doi.org/10.1016/j.ajhg.2012.11.021 23332916 10.1016/j.ajhg.2012.11.021 PMC3567271

[mgg3392-bib-0147] Masmoudi, S. , Antonarakis, S. E. , Schwede, T. , Ghorbel, A. M. , Grati, M. , Pappasavas, M. P. , … Guipponi, M. (2001). Novel missense mutations of TMPRSS3 in two consanguineous Tunisian families with non‐syndromic autosomal recessive deafness. Human Mutation, 18(2), 101–108. https://doi.org/10.1002/(ISSN)1098-1004 11462234 10.1002/humu.1159

[mgg3392-bib-0148] Masmoudi, S. , Charfedine, I. , Hmani, M. , Grati, M. , Ghorbel, A. M. , Elgaied‐Boulila, A. , … Ayadi, H. (2000). Pendred syndrome: Phenotypic variability in two families carrying the same PDS missense mutation. American Journal of Medical Genetics, 90(1), 38–44. https://doi.org/10.1002/(ISSN)1096-8628 10602116

[mgg3392-bib-0149] Masmoudi, S. , Charfedine, I. , Rebeh, I. B. , Rebai, A. , Tlili, A. , Ghorbel, A. M. , … Ayadi, H. (2004). Refined mapping of the autosomal recessive non‐syndromic deafness locus DFNB13 using eight novel microsatellite markers. Clinical Genetics, 66(4), 358–364. https://doi.org/10.1111/j.1399-0004.2004.00311.x 15355440 10.1111/j.1399-0004.2004.00311.x

[mgg3392-bib-0150] Masmoudi, S. , Elgaied‐Boulila, A. , Kassab, I. , Ben Arab, S. , Blanchard, S. , Bouzouita, J. E. , … Ayadi, H. (2000). Determination of the frequency of connexin 26 mutations in inherited sensorineural deafness and carrier rates in the Tunisian population using DGGE. Journal of Medical Genetics, 37(11), E39 https://doi.org/10.1136/jmg.37.11.e39 11073548 10.1136/jmg.37.11.e39 PMC1734469

[mgg3392-bib-0151] Masmoudi, S. , Tlili, A. , Majava, M. , Ghorbel, A. M. , Chardenoux, S. , Lemainque, A. , … Ayadi, H. (2003). Mapping of a new autosomal recessive nonsyndromic hearing loss locus (DFNB32) to chromosome 1p13.3‐22.1. European Journal of Human Genetics, 11(2), 185–188. https://doi.org/10.1038/sj.ejhg.5200934 12634867 10.1038/sj.ejhg.5200934

[mgg3392-bib-0152] Matisoo‐Smith, E.A. , Gosling, A.L. , Boocock, J. , Kardailsky, O. , Kurumilian, Y. , Roudesli‐Chebbi, S. , … Zalloua, P.A . (2016). A European mitochondrial haplotype identified in ancient phoenician remains from Carthage, North Africa. PLoS One 11(5): e0155046. https://doi.org/10.1371/journal.pone.0155046 eCollection 2016. 10.1371/journal.pone.0155046 PMC4880306 27224451

[mgg3392-bib-0153] M'Dimegh, S. , Aquaviva‐Bourdain, C. , Omezzine, A. , M'Barek, I. , Souche, G. , Zellama, D. , … Bouslama, A. (2016). A novel mutation in the AGXT gene causing primary hyperoxaluria type I: Genotype‐phenotype correlation. Journal of Genetics, 95(3), 659–666. https://doi.org/10.1007/s12041-016-0676-4 27659337 10.1007/s12041-016-0676-4

[mgg3392-bib-0154] M'Dimegh, S. , Omezzine, A. , M'barek, I. , Moussa, A. , Mabrouk, S. , Kaarout, H. , … Bouslama, A . (2017). Mutational analysis of Agxt in Tunisian population with primary hyperoxaluria type 1. Annals of Human Genetics, 81(1), 1–10. https://doi.org/10.1111/ahg.12178 27935012 10.1111/ahg.12178

[mgg3392-bib-0155] Messaoud, O. , Ben Rekaya, M. , Cherif, W. , Talmoudi, F. , Boussen, H. , Mokhtar, I. , … Zghal, M. (2010). Genetic homogeneity of mutational spectrum of group‐A Xeroderma pigmentosum in Tunisian patients. International Journal of Dermatology, 49, 544–548. https://doi.org/10.1111/j.1365-4632.2010.04421.x 20534089 10.1111/j.1365-4632.2010.04421.x

[mgg3392-bib-0156] Messaoud, O. , Ben Rekaya, M. , Jerbi, M. , Ouertani, I. , Kefi, R. , Laroussi, N. , … Abdelhak, S. (2013). The experience of a Tunisian referral centre in prenatal diagnosis of Xeroderma pigmentosum. Public Health Genomics, 16(5), 251–254. https://doi.org/10.1159/000354584 24021614 10.1159/000354584

[mgg3392-bib-0157] Messaoud, O. , Ben Rekaya, M. , Kefi, R. , Chebel, S. , Boughammoura‐Bouatay, A. , Bel Hadj Ali, H. , … Zghal, M . (2010). Identification of a primarily neurological phenotypic expression of Xeroderma pigmentosum complementation group A in a Tunisian family. British Journal of Dermatology, 162, 883–886. 20199544 10.1111/j.1365-2133.2010.09646.x

[mgg3392-bib-0158] Messaoud, O. , Ben Rekaya, M. , Ouragini, H. , Benfadhel, S. , Azaiez, H. , Kefi, R. , … Abdelhak, S. (2012). Severe phenotypes in two Tunisian families with novel XPA mutations: Evidence for a correlation between mutation location and disease severity. Archives of Dermatological Research, 304, 171–176. https://doi.org/10.1007/s00403-011-1190-4 22081045 10.1007/s00403-011-1190-4

[mgg3392-bib-0159] Messaoud, T. , Hadj Fredj, S. , Bibi, A. , Elion, J. , Ferec, C. , & Fattoum, S . (2005). Epidémiologie moléculaire de la mucoviscidose en Tunisie. Annales de Biologie Clinique, 63(6), 627–630. Article in french 16330381

[mgg3392-bib-0160] Messaoud, O. , Laroussi, N. , Ben Rekaya, M. , Jones, M. , Zghal, M. , Yacoub‐youssef, H. , … Abdelhak, S. (2013). Novel mutation in POLH gene responsible of severe phenotype of XP‐V. Clinical Dermatology, 1, 125–129.

[mgg3392-bib-0161] Methlouthi, J. , Mahdhaoui, N. , Bellaleh, M. , Guith, A. , Zouari, D. , Ayech, H. , … Séboui, H. (2016). Incidence of congenital heart disease in newborns after pulse oximetry screening introduction. Tunis Med, 94(3), 231–234. 27575509

[mgg3392-bib-0162] M'hamdi, O. , Ouertani, I. , Maazoul, F. , & Chaabouni, H . (2011). Prevalence of Bardet‐Biedl syndrome in Tunisia. Journal of Community Genetics, 2, 97–99. https://doi.org/10.1007/s12687-011-0040-6 22109794 10.1007/s12687-011-0040-6 PMC3186025

[mgg3392-bib-0163] M'hamdi, O. , Redin, C. , Stoetzel, C. , Ouertani, I. , Chaabouni, M. , Maazoul, F. , … Chaabouni, H . (2014). Clinical and genetic characterization of Bardet‐Biedl syndrome in Tunisia: Defining a strategy for molecular diagnosis. Clinical Genetics, 85 (2), 172–177. https://doi.org/10.1111/cge.12129 23432027 10.1111/cge.12129

[mgg3392-bib-0164] Morales, J. , Mulazzani, S. , Belhouchet, L. , Zazzo, A. , Berrio, L. , Eddargach, W. , … Peña‐Chocarro, L. (2015). First preliminary evidence for basketry and nut consumption in the Capsian culture (ca. 10,000–7500 BP): Archaeobotanical data from new excavations at El Mekta, Tunisia. Journal of Anthropological Archaeology, 37, 128–139. https://doi.org/10.1016/j.jaa.2014.12.005

[mgg3392-bib-0165] Morel, J. P . (2011). Les Fouilles de Byrsa (Secteur B) a Carthage. Comptes Rendus des Seances, 1, 325–363. (Article in French)

[mgg3392-bib-0166] Mosrati, A. M. , Schrauwen, I. , Ben Saiid, M. , Aifa‐Hmani, M. , Fransen, E. , Mneja, M. , … Masmoudi, S. (2013). Genome‐wide analysis reveals a novel autosomal‐recessive hearing loss locus DFNB80 on chromosome 2p16.1‐p21. Journal of Human Genetics, 58(2), 98–101. https://doi.org/10.1038/jhg.2012.141 23235334 10.1038/jhg.2012.141

[mgg3392-bib-0167] Mrissa, N. , Belal, S. , Hamida, C. B. , Amouri, R. , Turki, I. , Mrissa, R. , … Hentati, F. (2000). Linkage to chromosome 13q11‐12 of an autosomal recessive cerebellar ataxia in a Tunisian family. Neurology, 54(7), 1408–1414. https://doi.org/10.1212/WNL.54.7.1408 10751248 10.1212/wnl.54.7.1408

[mgg3392-bib-0168] Nagara, M. , Voskarides, K. , Nouira, S. , Ben Halim, N. , Kefi, R. , Aloulou, H. , … Abdelhak, S. (2014). Molecular investigation of distal renal tubular acidosis in Tunisia, evidence for founder mutations. Genet Test Mol Biomarkers, 18(11), 741–748. https://doi.org/10.1089/gtmb.2014.0175 25285676 10.1089/gtmb.2014.0175 PMC4217022

[mgg3392-bib-0169] Naouali, C. , Jones, M. , Nabouli, I. , Jerbi, M. , Tounsi, H. , Ben Rekaya, M. , … Yacoub‐Youssef, H. (2017). Epidemiological trends and clinicopathological features of cutaneous melanoma in sporadic and xeroderma pigmentosum Tunisian patients. International Journal of Dermatology, 56(1), 40–48. https://doi.org/10.1111/ijd.13448 27785785 10.1111/ijd.13448

[mgg3392-bib-0170] Nasrallah, F. , Hammami, M. B. , Ben Rhouma, H. , Fradj, S. H. , Azzouz, H. , Omar, S. , … Kaabachi, N. (2015). Clinical and biochemical profile of Tyrosinemia type 1 in Tunisia. Clinical Laboratory, 61(5–6), 487–492. 26118180 10.7754/clin.lab.2014.141009

[mgg3392-bib-0171] Nasri, K. , Ben Fradj, M. K. , Hamdi, T. , Aloui, M. , Ben Jemaa, N. , Nahdi, S. , … Siala Gaigi, S. (2014). Epidemiology of neural tube defect subtypes in Tunisia, 1991–2011. Pathology, Research and Practice, 210(12), 944–952. https://doi.org/10.1016/j.prp.2014.06.027 10.1016/j.prp.2014.06.027 25110062

[mgg3392-bib-0172] Nasri, A. , Kacem, I. , Sidhom, Y. , Hizem, Y. , Ben Djebara, M. , Gargouri, A. , … Gouider, R . (2015). Charcot‐Marie‐Tooth disease: Clinical and genetic spectrum in a Tunisian series (P3.181). Neurology, 84(14 supplement). http://www.neurology.org/content/84/14_Supplement/P3.181

[mgg3392-bib-0173] Noguchi, S. , McNally, E. M. , Ben Othmane, K. , Hagiwara, Y. , Mizuno, Y. , Yoshida, M. , … Ozawa, E. (1995). Mutations in the dystrophin‐associated protein gamma‐sarcoglycan in chromosome 13 muscular dystrophy. Science, 270(5237), 819–822. https://doi.org/10.1126/science.270.5237.819 7481775 10.1126/science.270.5237.819

[mgg3392-bib-0174] Ouali, F. , Siala, H. , Bibi, A. , Hadj Fredj, S. , Dakhlaoui, B. , Othmani, R. , … Messaoud, T. (2016). Prenatal diagnosis of hemoglobinopathies in Tunisia: An 18 years of experience. International Journal of Laboratory Hematology, 38(3), 223–232. https://doi.org/10.1111/ijlh.12457 26993054 10.1111/ijlh.12457

[mgg3392-bib-0175] Ouertani, I. , Chaabouni, M. , Chelly, I. , Kraoua, L. , Maazoul, F. , Trabelsi, M. , … Bouhamed‐Chaabouni, H. (2014). Clinical and molecular cytogenetic study of 38 Williams‐Beuren Syndrome Tunisian patients. Open Journal of Genetics, 4, 385–391. https://doi.org/10.4236/ojgen.2014.45036

[mgg3392-bib-0176] Oueslati, S. , Hadj Fredj, S. , Belhaj, R. , Siala, H. , Bibi, A. , & Messaoudi, T. (2015). Preliminary study of haplotypes linked to the rare cystic fibrosis E1104X mutation. Acta Physiologica Hungarica, 102(1), 86–93. https://doi.org/10.1556/APhysiol.101.2014.013 25481366 10.1556/APhysiol.101.2014.013

[mgg3392-bib-0177] Ouesleti, S. , Brunel, V. , Ben Turkia, H. , Dranguet, H. , Miled, A. , Miladi, N. , … Bekri, S. (2011). Molecular characterization of MPS IIIA, MPS IIB and MPS IIIC in Tunisian patients. Clinica Chimica Acta, 412, 2326–2331. https://doi.org/10.1016/j.cca.2011.08.032 10.1016/j.cca.2011.08.032 21910976

[mgg3392-bib-0178] Quint, A. , Lerer, I. , Sagi, M. , & Abeliovich, D. (2005). Mutation spectrum in Jewish cystic fibrosis patients in Israel: Implication to carrier screening. American Journal of Medical Genetics. Part A, 136(3), 246–248. https://doi.org/10.1002/(ISSN)1552-4833 15948195 10.1002/ajmg.a.30823

[mgg3392-bib-0179] Rahmani, N. (2004). Technological and cultural change among the last hunter‐gatherers of the Maghreb: The Capsian (10,000–6000 B.P.). Journal of World Prehistory, 18, 57–106. https://doi.org/10.1023/B:JOWO.0000038658.50738.eb

[mgg3392-bib-0180] Riahi, Z. , Bonnet, C. , Zainine, R. , Lahbib, S. , Bouyacoub, Y. , Bechraoui, R. , … Petit, C . (2015). Whole exome sequencing identifies mutations in Usher syndrome genes in profoundly deaf Tunisian patients. PLoS ONE, 10(3), e0120584. https://doi.org/10.1371/journal.pone.0120584 eCollection 2015. 10.1371/journal.pone.0120584 PMC4370767 25798947

[mgg3392-bib-0181] Riahi, Z. , Bonnet, C. , Zainine, R. , Louha, M. , Bouyacoub, Y. , Laroussi, N. , … Petit, C. (2014). Whole exome sequencing identifies new causative mutations in Tunisian families with non‐syndromic deafness. PLoS ONE, 9(6), e99797 https://doi.org/10.1371/journal.pone.0099797 24926664 10.1371/journal.pone.0099797 PMC4057390

[mgg3392-bib-0182] Riahi, A. , Chabouni‐Bouhamed, H. , & Kharrat, M. (2017). Prevalence of BRCA1 and BRCA2 large genomic rearrangements in Tunisian high‐risk breast/ovarian cancer families: Implications for genetic testing. Cancer Genet, 210, 22–27. https://doi.org/10.1016/j.cancergen.2016.11.002 28212807 10.1016/j.cancergen.2016.11.002

[mgg3392-bib-0183] Riahi, Z. , Hammami, H. , Ouragini, H. , Messai, H. , Zainine, R. , Bouyacoub, Y. , … Abdelhak, S. (2013). Update of the spectrum of GJB2 gene mutations in Tunisian families with autosomal recessive nonsyndromic hearing loss. Gene, 525, 1–4. https://doi.org/10.1016/j.gene.2013.04.078 23680645 10.1016/j.gene.2013.04.078

[mgg3392-bib-0184] Riahi, Z. , Zainine, R. , Mellouli, Y. , Hannachi, R. , Bouyacoub, Y. , Laroussi, N. , … Besbes, G. (2013). Compound heterozygosity for dominant and recessive GJB2 mutations in a Tunisian family and association with successful cochlear implant outcome. International Journal of Pediatric Otorhinolaryngology, 77(9), 1481–1484. https://doi.org/10.1016/j.ijporl.2013.06.013 23856378 10.1016/j.ijporl.2013.06.013

[mgg3392-bib-0185] Riou, S. , el Younsi, C. , & Chaabouni, H. (1989). Consanguinity in the population of northern Tunisia. Tunis Med, 67, 167–172. 2756579

[mgg3392-bib-0186] Romdhane, L. , & Abdelhak, S. (2011). Genetic diseases in the Tunisian population. American Journal of Medical Genetics. Part A, 155A(1), 238–267. https://doi.org/10.1002/ajmg.a.33771. Review 21204241 10.1002/ajmg.a.33771

[mgg3392-bib-0187] Romdhane, L. , Kefi, R. , Azaiez, H. , Ben Halim, N. , Dellagi, K. , & Abdelhak, S. (2012). Founder mutations in Tunisia: Implications for diagnosis in North Africa and Middle East. Orphanet Journal of Rare Diseases, 7, 52 https://doi.org/10.1186/1750-1172-7-52 22908982 10.1186/1750-1172-7-52 PMC3495028

[mgg3392-bib-0188] Romdhane, L. , Messaoud, O. , Bouyacoub, Y. , Kerkeni, E. , Naouali, C. , Cherif Ben Abdallah, L. , … Abdelhak, S . (2016). Comorbidity in the Tunisian population. Clinical Genetics, 89(3), 312–319. https://doi.org/10.1111/cge.12616 26010040 10.1111/cge.12616

[mgg3392-bib-0189] Sahli, C. A. , Ben Salem, I. , Jouini, L. , Laouini, N. , Dabboubi, R. , Hadj Fredj, S. , … Messaoud, T. (2016). Setup of a protocol of molecular diagnosis of β‐Thalassemia mutations in Tunisia using denaturing high‐performance liquid chromatography (DHPLC). Journal of Clinical Laboratory Analysis, 30(5), 392–398. https://doi.org/10.1002/jcla.21867 27086580 10.1002/jcla.21867 PMC6807109

[mgg3392-bib-0190] Sanchez‐Mazas, A . (2000). *Chap 4:* The Berbers of North Africa: Genetic relationships according to HLA and other polymorphisms In Arnaiz‐Villena A., Martinez G. & Gomez‐Casado E. (Ed.), Prehistoric Iberia: Genetics, anthropology, and Linguistics (pp. 65–77). New‐York: Kluwer Academic/Plenum Publishers https://doi.org/10.1007/978-1-4615-4231-5

[mgg3392-bib-0191] Shaub, K . (2016). Special Education in Tunisia: A Case Study in Social Entrepreneurship (2016). *Independent Study Project (ISP) Collection*, 2376. http://digitalcollections.sit.edu/isp_collection/2376

[mgg3392-bib-0192] Smaoui, N. , Chaabouni, M. , Sergeev, Y. V. , Kallel, H. , Li, S. , Mahfoudh, N. , … Hejtmancik, J. F. (2006). Screening of the eight BBS genes in Tunisian families: No evidence of triallelism. Investigative Ophthalmology & Visual Science, 47, 3487–3495. https://doi.org/10.1167/iovs.05-1334 16877420 10.1167/iovs.05-1334

[mgg3392-bib-0193] Tadmouri, G. O. , Al Ali, M. T. , Al‐Haj Ali, S. , & Al Khaja, N . (2006). CTGA: The database for genetic disorders in Arab populations. Nucleic Acids Research, 34 (Database issue), D602–D606. https://doi.org/10.1093/nar/gkj015 16381941 10.1093/nar/gkj015 PMC1347378

[mgg3392-bib-0194] Tadmouri, G. O. , Sastry, K. S. , & Chouchane, L. (2014). Arab gene geography: From population diversities to personalized medical genomics. Global Cardiology Science & Practice, 4, 394–408. https://doi.org/10.5339/gcsp.2014.54. eCollection 2014. Review 10.5339/gcsp.2014.54 PMC4355514 25780794

[mgg3392-bib-0195] Talmoudi, F. , Kilani, O. , Ayed, W. , Ben Halim, N. , Mellouli, F. , Torjmane, L. , … Amouri, A. (2013). Differentiation of Fanconi anemia and aplastic anemia using mitomycin C test in Tunisia. C.R. Biologies, 336(1), 29–33. https://doi.org/10.1016/j.crvi.2013.02.001 23537767 10.1016/j.crvi.2013.02.001

[mgg3392-bib-0196] Tamary, H. , Bar‐Yam, R. , Shalmon, L. , Rachavi, G. , Krostichevsky, M. , Elhasid, R. , … Zaizov, R. (2000). Fanconi anaemia group A (FANCA) mutations in Israeli non‐Ashkenazi Jewish patients. British Journal of Haematology, 111(1), 338–343. https://doi.org/10.1046/j.1365-2141.2000.02323.x 11091222 10.1046/j.1365-2141.2000.02323.x

[mgg3392-bib-0197] Tlili, A. , Charfedine, I. , Lahmar, I. , Benzina, Z. , Mohamed, B. A. , Weil, D. , … Ayadi, H. (2005). Identification of a novel frameshift mutation in the DFNB31/WHRN gene in a Tunisian consanguineous family with hereditary non‐syndromic recessive hearing loss. Human Mutation, 25(5), 503 https://doi.org/10.1002/(ISSN)1098-1004 10.1002/humu.9333 15841483

[mgg3392-bib-0198] Tlili, A. , Männikkö, M. , Charfedine, I. , Lahmar, I. , Benzina, Z. , Ben Amor, M. , … Ayadi, H. (2005). A novel autosomal recessive non‐syndromic deafness locus, DFNB66, maps to chromosome 6p21.2–22.3 in a large Tunisian consanguineous family. Human Heredity, 60, 123–128. https://doi.org/10.1159/000088974 16244493 10.1159/000088974

[mgg3392-bib-0199] Tlili, A. , Masmoudi, S. , Dhouib, H. , Bouaziz, S. , Rebeh, I. B. , Chouchen, J. , … Ayadi, H. (2007). Localization of a novel autosomal recessive non‐syndromic hearing impairment locus DFNB63 to chromosome 11q13.3–q13.4. Annals of Human Genetics, 71, 271–275. https://doi.org/10.1111/j.1469-1809.2006.00337.x 17166180 10.1111/j.1469-1809.2006.00337.x

[mgg3392-bib-0200] Tlili, A. , Rebeh, I. B. , Aifa‐Hmani, M. , Dhouib, H. , Moalla, J. , Tlili‐Chouchène, J. , … Masmoudi, S. (2008). TMC1 but not TMC2 is responsible for autosomal recessive nonsyndromic hearing impairment in Tunisian families. Audiol Neurootol, 13(4), 213–218. https://doi.org/10.1159/000115430 18259073 10.1159/000115430

[mgg3392-bib-0201] Toledano‐Alhadef, H. , Basel‐Vanagaite, L. , Magal, N. , Davidov, B. , Ehrlich, S. , Drasinover, V. , … Shohat, M. (2001). Fragile‐X carrier screening and the prevalence of premutation and full‐mutation carriers in Israel. American Journal of Human Genetics, 69(2), 351–360. https://doi.org/10.1086/321974 11443541 10.1086/321974 PMC1235307

[mgg3392-bib-0202] Trabelsi, M. , Bahri, W. , Habibi, M. , Zainine, R. , Maazoul, F. , Ghazi, B. , … Mrad, R. (2013). GJB2 and GJB6 screening in Tunisian patients with autosomal recessive deafness. International Journal of Pediatric Otorhinolaryngology, 77(5), 714–716. https://doi.org/10.1016/j.ijporl.2013.01.024 23434199 10.1016/j.ijporl.2013.01.024

[mgg3392-bib-0203] Trinh, J. , Amouri, R. , Duda, J. E. , Morley, J. F. , Read, M. , Donald, A. , … Farrer, M. J. (2014). Comparative study of Parkinson's disease and leucine‐rich repeat kinase 2 p. G2019S parkinsonism. Neurobiology of Aging, 35(5), 1125–1131. https://doi.org/10.1016/j.neurobiolaging.2013.11.015 24355527 10.1016/j.neurobiolaging.2013.11.015

[mgg3392-bib-0204] Trinh, J. , Gustavsson, E. K. , Vilariño‐Güell, C. , Bortnick, S. , Latourelle, J. , McKenzie, M. B. , … Farrer, M. J. (2016). DNM3 and genetic modifiers of age of onset in LRRK2 Gly2019Ser parkinsonism: A genome‐wide linkage and association study. Lancet Neurol, 15(12), 1248–1256. https://doi.org/10.1016/S1474-4422(16)30203-4 27692902 10.1016/S1474-4422(16)30203-4

[mgg3392-bib-0205] Troudi, W. , Uhrhammer, N. , Romdhane, K. B. , Sibille, C. , Amor, M. B. , Khodjet El Khil, H. , … Elgaaied, A. B. (2008). Complete mutation screening and haplotype characterization of BRCA1 gene in Tunisian patients with familial breast cancer. Cancer Biomark, 4(1), 11–18. https://doi.org/10.3233/CBM-2008-4102 18334730 10.3233/cbm-2008-4102

[mgg3392-bib-0206] Turchi, C. , Buscemi, L. , Giacchino, E. , Onofri, V. , Fendt, L. , Parson, W. , & Tagliabracci, A. (2009). Polymorphisms of mtDNA control region in Tunisian and Moroccan populations: An enrichment of forensic mtDNA databases with Northern Africa data. Forensic Science International. Genetics., 3, 166–172. https://doi.org/10.1016/j.fsigen.2009.01.014 19414164 10.1016/j.fsigen.2009.01.014

[mgg3392-bib-0207] Warren, L. , Gibson, R. , Ishihara, L. , Elango, R. , Xue, Z. , Akkari, A. , … Hentati, F. (2008). A founding LRRK2 haplotype shared by Tunisian, US, European and Middle Eastern families with Parkinson's disease. Parkinsonism & Related Disorders, 14, 77–80. https://doi.org/10.1016/j.parkreldis.2007.02.001 17433753 10.1016/j.parkreldis.2007.02.001

[mgg3392-bib-0208] Weil, D. , El‐Amraoui, A. , Masmoudi, S. , Mustapha, M. , Kikkawa, Y. , Lainé, S. , … Petit, C. (2003). Usher syndrome type I G (USH1G) is caused by mutations in the gene encoding SANS, a protein that associates with the USH1C protein, harmonin. Human Molecular Genetics, 5, 463–471. https://doi.org/10.1093/hmg/ddg051 10.1093/hmg/ddg051 12588794

[mgg3392-bib-0209] Weil, D. , Küssel, P. , Blanchard, S. , Lévy, G. , Levi‐Acobas, F. , Drira, M. , … Petit, C. (1997). The autosomal recessive isolated deafness, DFNB2, and the Usher 1B syndrome are allelic defects of the myosin‐VIIA gene. Nature Genetics, 16, 191–193. https://doi.org/10.1038/ng0697-191 9171833 10.1038/ng0697-191

[mgg3392-bib-0210] Weinstein, M. , Eisensmith, R. C. , Abadie, V. , Avigad, S. , Lyonnet, S. , Schwartz, G. , … Shiloh, Y. (1993). A missense mutation, S349P, completely inactivates phenylalanine hydroxylase in North African Jews with phenylketonuria. Human Genetics, 90(6), 645–649. 8095248 10.1007/BF00202483

[mgg3392-bib-0211] Zalloua, P. A. , Platt, D. E. , El Sibai, M. , Khalife, J. , Makhoul, N. , Haber, M. , … Tyler‐Smith, C. (2008). Genographic Consortium. Identifying genetic traces of historical expansions: Phoenician footprints in the Mediterranean. American Journal of Human Genetics, 83(5), 633–642. https://doi.org/10.1016/j.ajhg.2008.10.012 18976729 10.1016/j.ajhg.2008.10.012 PMC2668035

[mgg3392-bib-0212] Zghal, M. , Fazaa, B. , & Kamoun, M. R. (2006). Xeroderma pigmentosum. EMC (Elsevier SAS, Paris). Dermatologie 10, 98–660‐A‐10. Paris: Elsevier.

[mgg3392-bib-0213] Zghal, M. , Fazaa, B. , Zghal, A. , Mokhtar, I. , Sarasin, A. , & Kamoun, M.R. , & Gharbi, M.R. (2003) [A whole family affected by xeroderma pigmentosum: Clinical and genetic particularities]. Annales de Dermatologie et de Venereologie, 130(1 Pt1), 31–36. (Article in French) 12605154

